# Imaging of cranial nerves: a pictorial overview

**DOI:** 10.1186/s13244-019-0719-5

**Published:** 2019-03-15

**Authors:** Nicola Romano, Margherita Federici, Antonio Castaldi

**Affiliations:** 1 Department of Health Sciences (DISSAL) - Radiology Section, University of Genoa, Genoa, Italy; 2 Department of Diagnostic and Interventional Neuroradiology, E.O. Ospedali Galliera, Genoa, Italy

**Keywords:** Cranial nerves, Magnetic resonance imaging, Computed tomography, Anatomy, Pathology

## Abstract

The human body has 12 pairs of cranial nerves that control motor and sensory functions of the head and neck. The anatomy of cranial nerves is complex and its knowledge is crucial to detect pathological alterations in case of nervous disorders. Therefore, it is necessary to know the most frequent pathologies that may involve cranial nerves and recognize their typical characteristics of imaging. Cranial nerve dysfunctions may be the result of pathological processes of the cranial nerve itself or be related to tumors, inflammation, infectious processes, or traumatic injuries of adjacent structures. Magnetic resonance imaging (MRI) is considered the gold standard in the study of the cranial nerves. Computed tomography (CT) allows, usually, an indirect view of the nerve and is useful to demonstrate the intraosseous segments of cranial nerves, the foramina through which they exit skull base and their pathologic changes. The article is a complete pictorial overview of the imaging of cranial nerves, with anatomic and pathologic descriptions and great attention to illustrative depiction. We believe that it could be a useful guide for radiologists and neuroradiologists to review the anatomy and the most important pathologies that involve cranial nerves and their differential diagnosis.

## Key points

- Anatomy of cranial nerves is complex and its knowledge is crucial to detect pathological alterations in case of nervous disorders
- Magnetic resonance imaging (MRI) is the gold standard technique in the study of the cranial nerves
- Steady-state free procession (SSFP) images are the best sequences for the visualization of the cisternal segments showing dark cranial nerves against a background of bright cerebrospinal fluid (CSF)
- Computed tomography (CT) can be useful to evaluate intraosseous segments of cranial nerves, skull base foramina, and bony traumatic lesions
- Cranial nerve dysfunctions may be the result of pathological processes of the cranial nerve itself or may be associated with tumor, inflammation, infectious processes, or traumatic injuries of adjacent structures

## Introduction

The human body has 12 pairs of cranial nerves that control motor and sensory functions of the head and neck. This article provides a pictorial overview of the imaging of cranial nerves, with a special focus on their anatomy and pathology. Radiologists and, specially, neuroradiologists should be familiar with the anatomy of each cranial nerve and should know clinical and imaging findings of their dysfunctions.

## Imaging

**Fig. 1:**
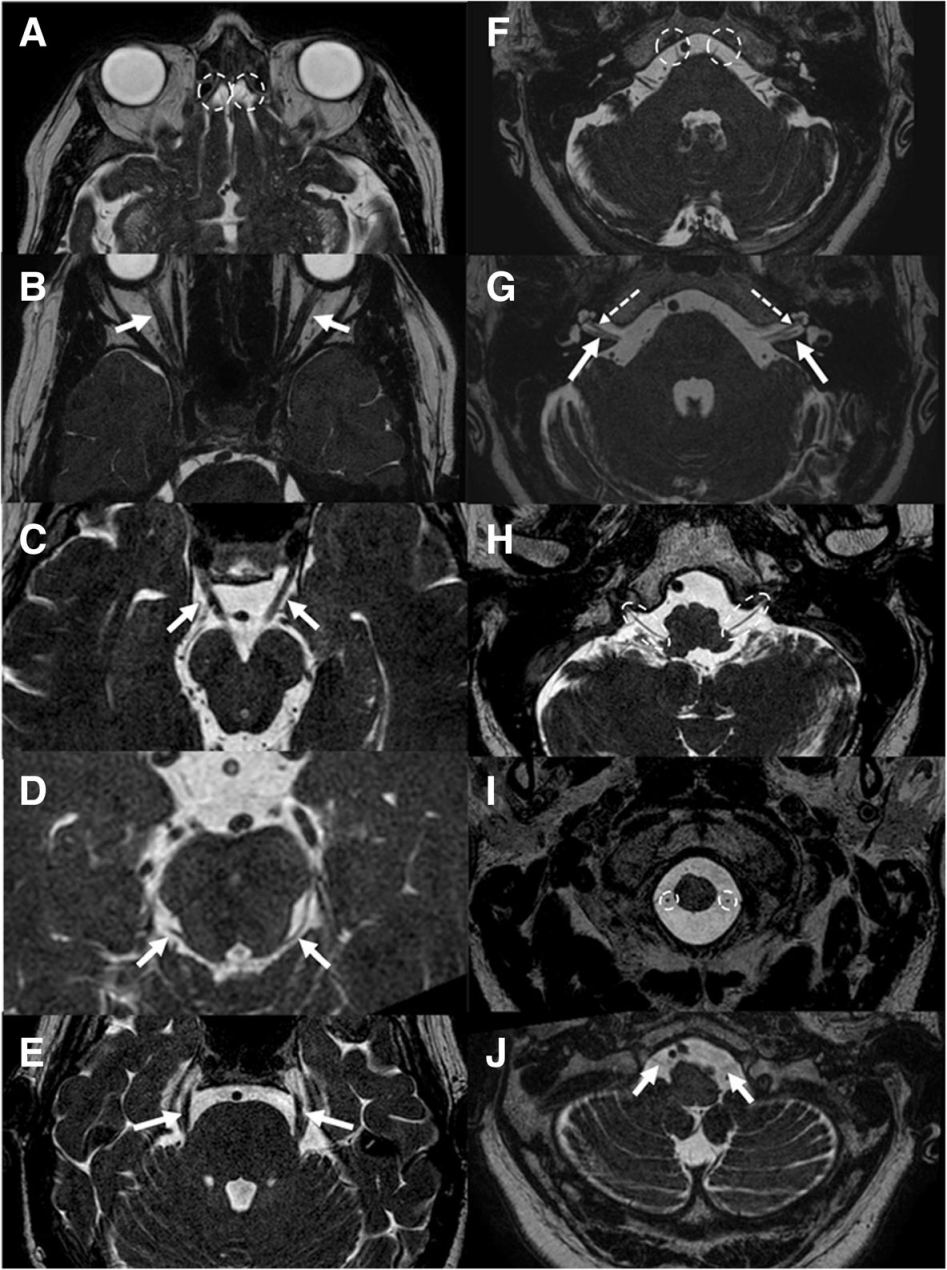
MRI of the cisternal tracts of normal cranial nerves. Steady-state free procession (SSFP), axial planes. **a** Olfactory (circles); **b** optic (arrows); **c** oculomotor (arrows); **d** trochlear (arrows); **e** trigeminal (arrows); **f** abducens (circles); **g** facial (thin and dotted arrows) and vestibulocochlear (thick arrows); **h** glossopharyngeal and vagus nerve (circles); **i** spinal accessory (circles); **j** hypoglossal (arrows)

**Fig. 2:**
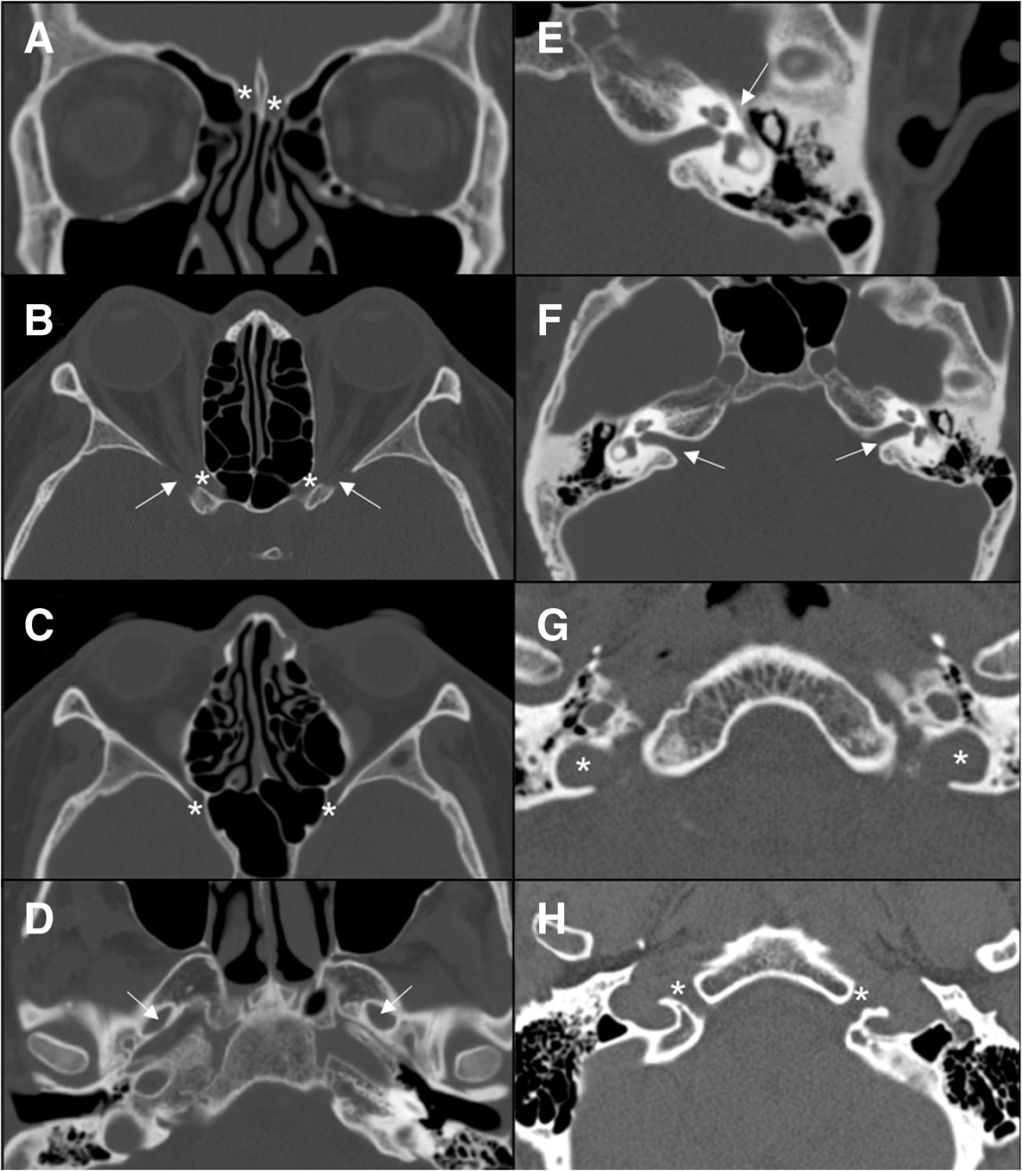
CT aspect of the most important foramina of the skull base. **a** Cribriform plate (asterisk); **b** optic canal (asterisk) and superior orbital fissure (arrow); **c** foramen rotundum (asterisk); **d** foramen ovale (arrow); **e** facial canal (arrow); **f** internal auditory meatus (arrow); **g** jugular foramen (asterisk); **h** hypoglossal canal (asterisk)

MRI is considered the gold standard in the study of cranial nerves. Table 1 summarizes the most important sequences and features in their study. Several scientific articles have underlined the importance of SSFP sequences for the visualization of the cisternal spaces of cranial nerves thanks to their sub-millimetric spatial and high contrast resolution [1–4]. These sequences typically show dark cranial nerves against a background of bright CSF [2, 5] (Fig. 1). The enhancement of the nerve, after gadolinium administration, is associated to disruption of the blood-nerve barrier and may be secondary to neoplasm, inflammation, demyelination, ischemia, trauma, radiation treatment, and axonal degeneration. Three-dimensional T1-weighted gradient echo sequences can provide a better visualization of the nerve enhancement, surrounded by CSF. MRI could also be useful to assess denervation changes of “end-organ muscles” (Table 2) [5]. CT is inferior to MRI in the visualization of the cranial nerves themselves, due to its low contrast resolution. Therefore, it can be useful to evaluate the intraosseous segments of cranial nerves and the possible associated bony changes. Particularly, CT is optimal to study the bony foramina of the skull base (Table 3, Fig. 2) and bony traumatic lesions [6].

**Table 1:** Utility of MRI sequences for the study of cranial nerves

MRI sequences	Features
T1-weighted sequences	- Anatomical definition - Invasion of fat planes - Denervation changes
T2-weighted sequences	- Lesion characterization - Patency of CSF spaces - Denervation changes
Fast spin echo; steady-state free procession	- Cisternal course of the nerve - Neurovascular conflict
DWI–FLAIR sequences	- Ischemic lesion
Post-gadolinium T1-weighted sequences (eventually with fat-suppression)	- Enhancement of the nerve - Perineural spread - Meningeal infiltration

**Table 2:** Summary of the most important “end-organ muscles”

Nerve	Muscles to evaluate for denervation
III	Superior, inferior, medial rectus, and inferior oblique muscles
IV	Superior oblique muscle
V	Muscles of mastication: masseter, temporalis, and pterygoid (V3)
VI	Lateral rectus muscle
X	Vocal cords
XI	Sternocleidomastoid, trapezius muscles
XII	Tongue muscles

**Table 3:** Summary of the exits of cranial nerves from the skull

Nerve	Foramen
I	Cribriform plate
II	Optic canal
III	Superior orbital fissure
IV	Superior orbital fissure
V1	Superior orbital fissure
V2	Foramen rotundum
V3	Foramen ovale
VI	Superior orbital fissure
VII	Internal auditory meatus/facial canal
VIII	Internal auditory meatus
IX	Jugular foramen
X	Jugular foramen
XI	Jugular foramen
XII	Hypoglossal canal

## Anatomy

**Fig. 3:**
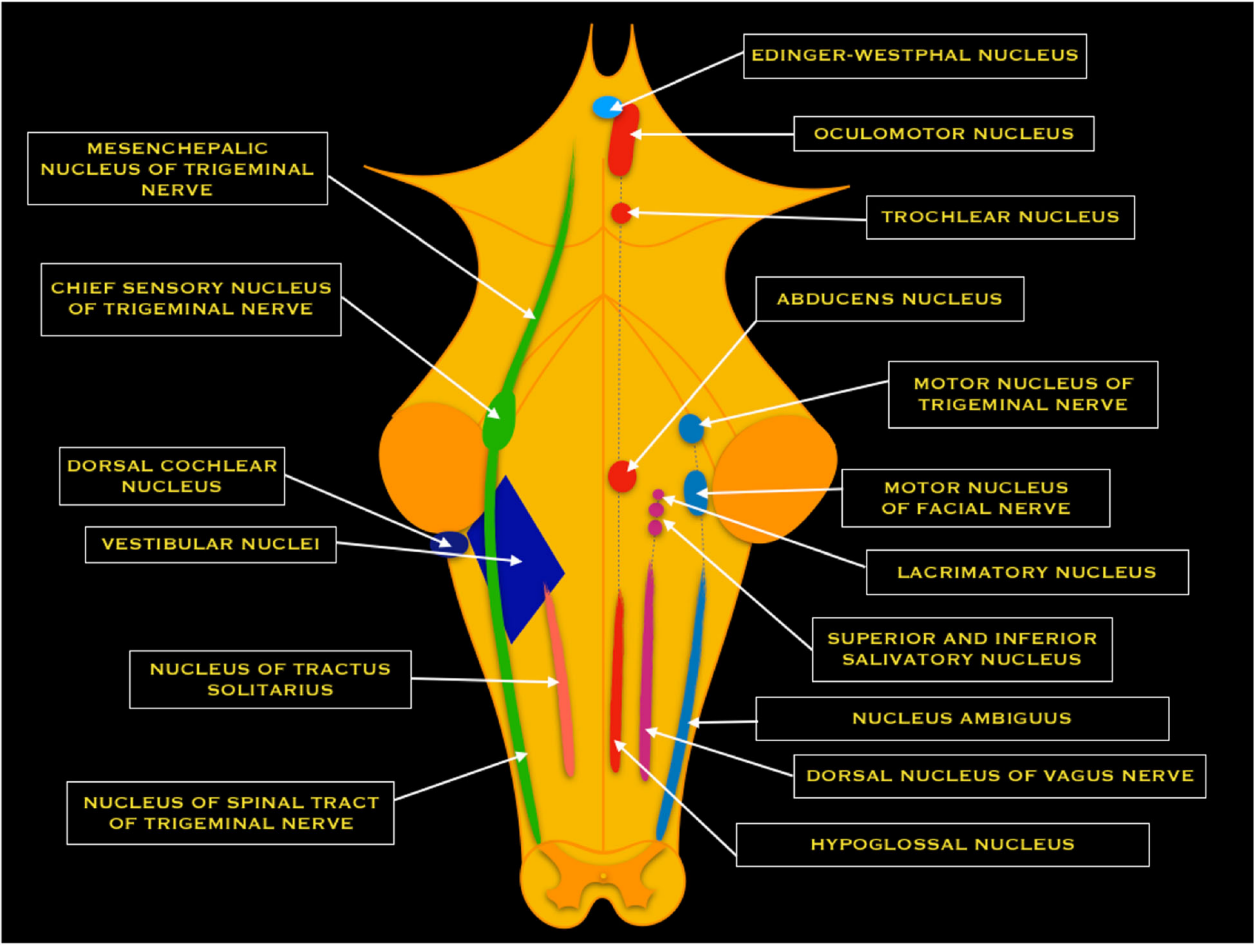
Schematic drawing of the nuclei of cranial nerves. The longitudinal view of the brainstem shows the position of the motor (right), sensory (left), and autonomic cell groups associated with cranial nerves. On the right, from the midline to outside: somatic motor column (motor somatic nuclei of the III, IV, VI, and XII cranial nerves) (red nuclei); parasympathetic column (derived from the III, VII, IX, and X cranial nerves) (purple nuclei); branchial motor column (motor nuclei of V, VII, IX, X, and XI cranial nerves) (light blue nuclei). On the left, from the midline to outside: visceral sensory column (IX, X cranial nerves) (pink); special somatic sensory column (VII, VIII, IX cranial nerves) (blue); general somatic sensory column (V, VII, IX, X cranial nerves) (green)

Nuclei of cranial nerves, except I and II, are located in the brainstem (Fig. 3); from their origin, nerves are divided in cisternal, intracranial, and extracranial segments.

Microscopically, cranial nerves are surrounded by a series of a connective tissue sheaths: endoneurium, perineurium, and epineurium. Axons of each nerve, except I and II, are myelinated by Schwann cells.

Functionally, they may have motor efferents and/or sensory afferents (Table 4)*.*

**Table 4:** Summary of efferents and afferents fibers of cranial nerves

Motor efferents	
General somatic efferents	III, IV, VI, XII
General visceral efferents	III, VII, IX, X
Special visceral efferents	V, VII, IX, X, XI
Sensory afferents	
General somatic afferents	V, VII, X
General visceral afferents	IX, X
Special afferents	I, II, VII, VIII, IX, X

### I cranial nerve - Olfactory nerve

**Fig. 4:**
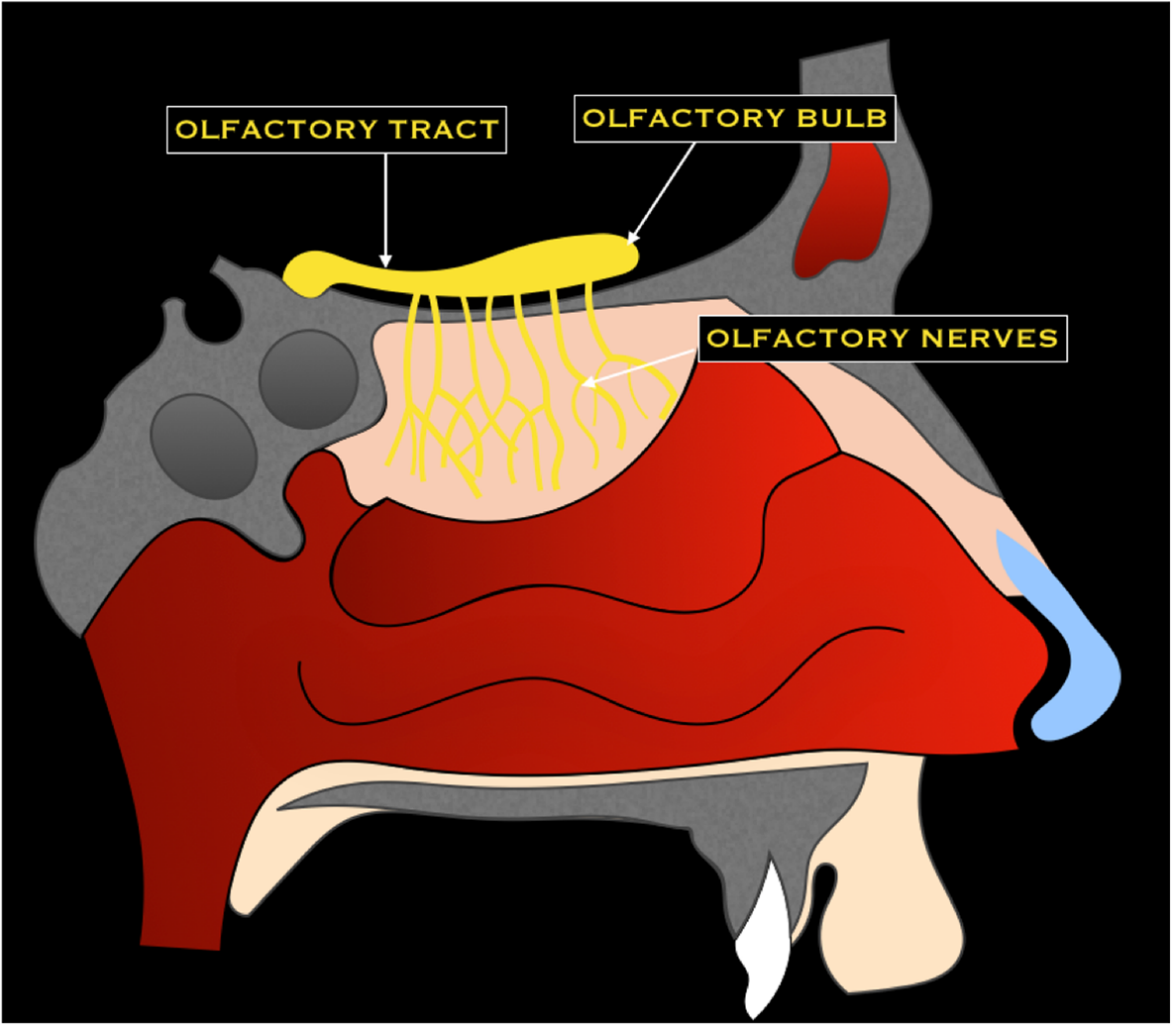
Schematic drawing of the olfactory nerve

The olfactory nerve is a sensitive nerve that conveys olfactory stimuli from the nasal cavity to the brain [7]. It consists of white matter tracts not surrounded by Schwann cells*.* Olfactory receptors are located in the mucosa of nasal cavity and olfactory filiae represent their axons. They enter the anterior cranial fossa through the cribriform plate and terminate in the olfactory bulb [6]. Successively, the nerve courses between the gyrus rectus and the medial orbital gyrus. The secondary axons terminate in the inferomedial temporal lobe, uncus, and entorhinal cortex [3] (Fig. 4).

### II cranial nerve - Optic nerve

**Fig. 5:**
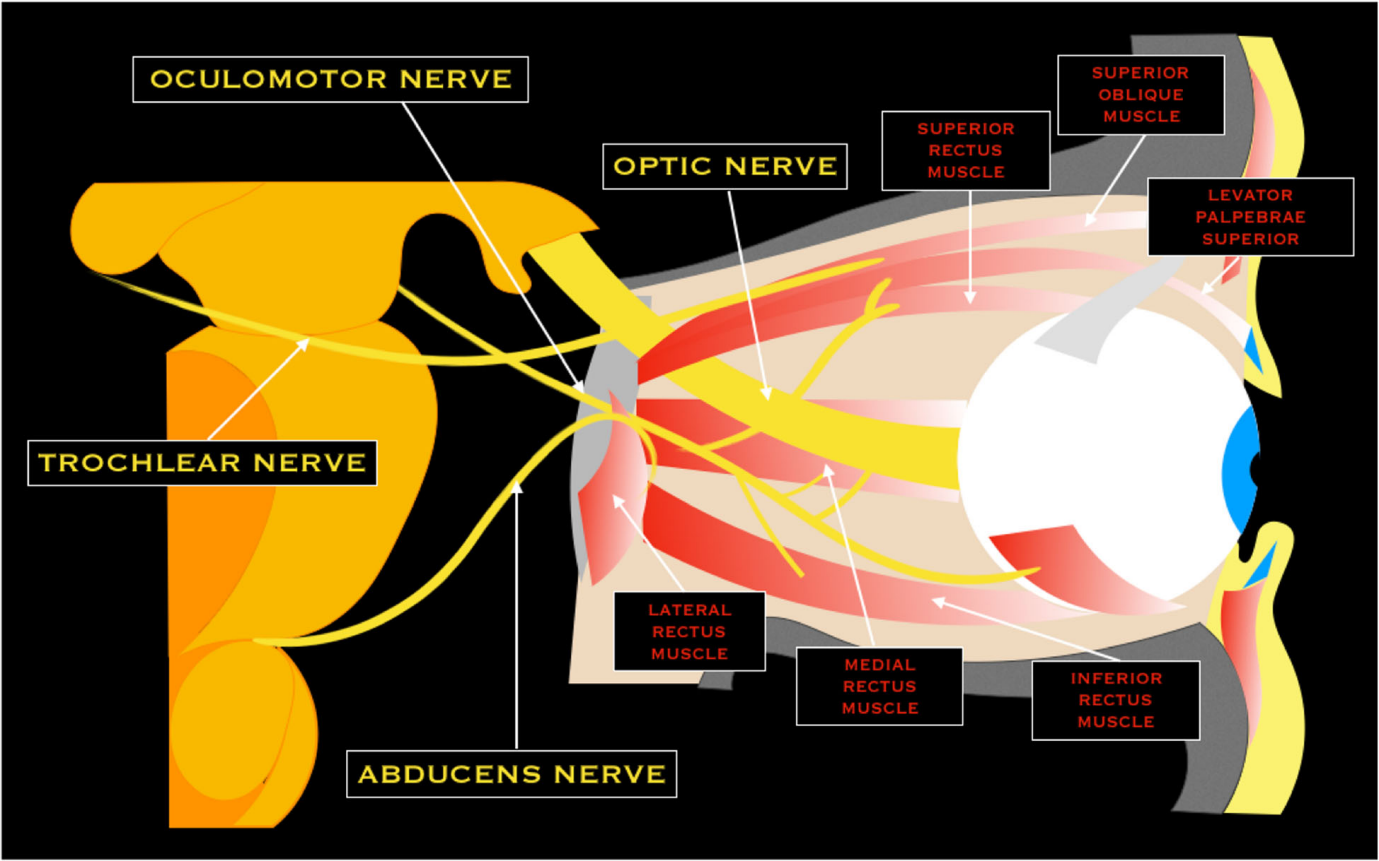
Schematic drawing of the optic and oculomotors nerves

Optic nerve represents an extension of central nervous system and is myelinated by oligodendrocytes [8]. Optic nerve emerges from the posterior pole of the ocular globe. It is approximately 50 mm in length, divided in four segments: intraocular (1 mm), intraorbital (25 mm), intracanalicular (9 mm), and prechiasmatic (16 mm) [8]. The nerve joins the contralateral to form the optic chiasma, where the nasal fibers from each nerve decussate, while the temporal fibers not. From the optic chiasm, optic tract courses posteriorly along the cerebral peduncles and synapses at the lateral geniculate nuclei. From the lateral geniculate body, optic radiation reaches the primary visual cortex in the occipital lobe (Brodmann area 17) [6] (Fig. 5).

### III cranial nerve - Oculomotor nerve

The oculomotor nerve has a somatic motor function of most ocular estrinsic muscles (inferior, superior, middle rectus, inferior oblique, and levator palpebrae superior muscles) and a parasympathetic function (ciliaris and sphincter pupillae muscles) [6]. Somatic motor fibers of oculomotor originate from the nuclear complex located in the midbrain at the level of the superior colliculi, ventral to the cerebral aqueduct and the periaqueductal gray matter. The parasympathetic fibers arise in the Edinger-Westphal nucleus, situated dorsally to the oculomotor nuclear complex. The nerve emerges from the interpeduncular cistern running through the perimesencephalic cistern, superiorly to the posterior cerebral artery (PCA) and inferiorly to the superior cerebellar artery (SCA) [6]. Successively, the nerve enters in the cavernous sinus where is the most superior nerve, to reach the orbit through the superior orbital fissure [3] (Fig. 5).

### IV cranial nerve - Trochlear nerve

The trochlear nerve innervates only the superior oblique muscle. It is the unique nerve with a root zone arising from the posterior brainstem where its nucleus lies [3]. After exiting the pons, the nerve curves over the superior cerebellar peduncle and then runs between the SCA and the PCA. Successively, it runs along the free margin of the tentorium and then enters the cavernous sinus, inferior to the III nerve and superior to the ophthalmic division of the V nerve [6]. Passing the cavernous sinus, the trochlear nerve enters the orbit through the superior orbital fissure [3] (Fig. 5).

### V cranial nerve - Trigeminal nerve

**Fig. 6:**
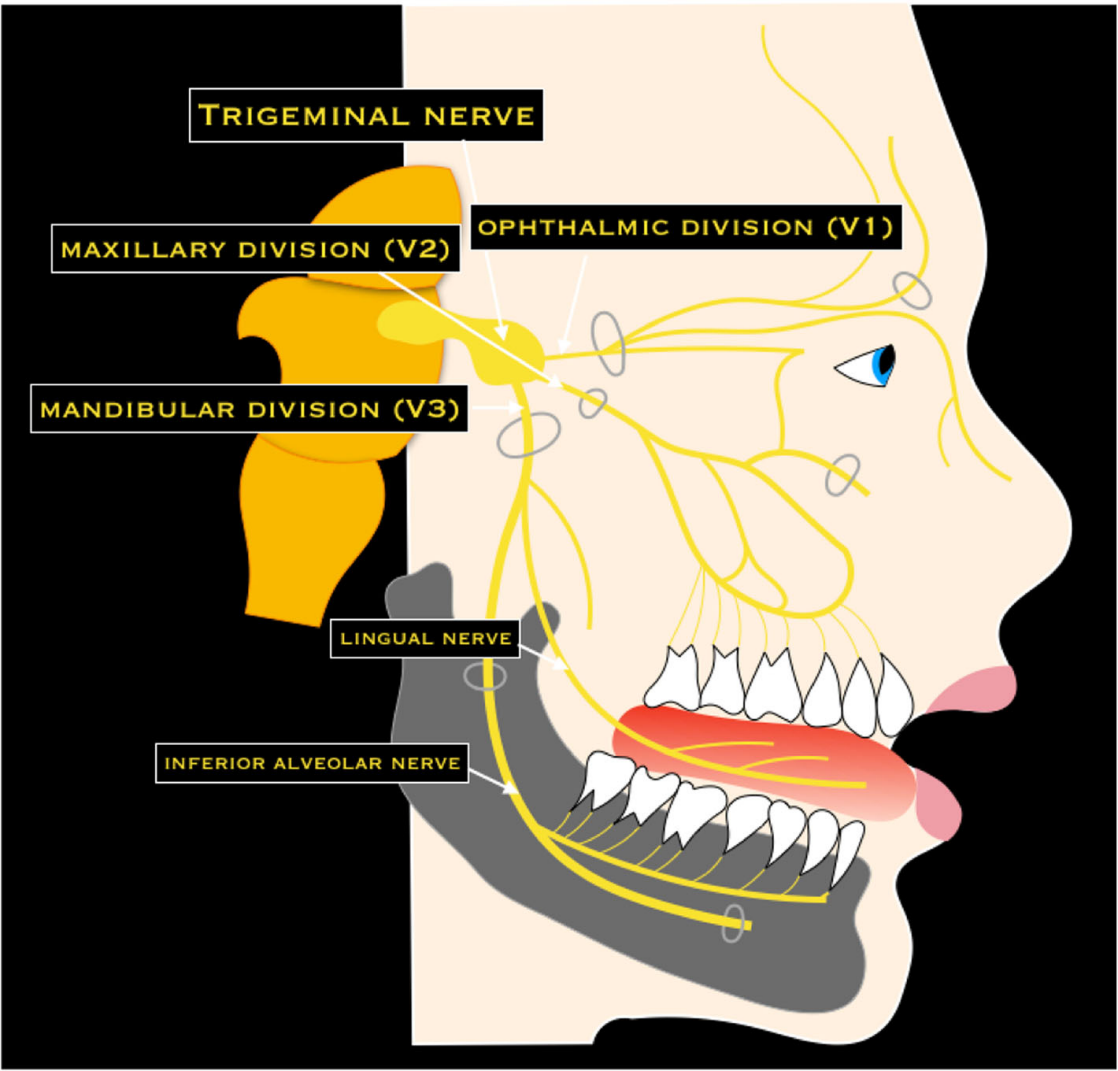
Schematic drawing of the trigeminal nerve

The trigeminal nerve is the largest cranial nerve, composed of sensory and motor roots [3]. Four trigeminal nuclei (spinal nucleus of V, principal sensory nucleus of V, motor nucleus of V, and mesencephalic nucleus) extend from the midbrain down to the upper cervical medulla [6]. The nerve emerges straight forward from the lateral pons and pass in the Meckel’s cave, where is located the trigeminal ganglion (Gasserian ganglion), from which the nerve splits into three subdivisions. The ophthalmic (V1) and the maxillary (V2) divisions pass in the lateral wall of cavernous sinus, inferior to the VI cranial nerve. The ophthalmic division exits the skull via the superior orbital fissure (with the III, IV, VI cranial nerves and the superior ophthalmic vein). The maxillary division exits the skull through the foramen rotundum and crosses the pterygopalatine fossa. Successively, it enters the orbits via the inferior orbital fissure, passes within the infraorbital groove, and reaches the face through the infraorbital foramen. The mandibular division (V3) exits the skull via the foramen ovale and passes in the infratemporal fossa, where it divides into some branches (meningeal branch, medial and lateral pterygoid nerves, masseteric nerve, deep temporal nerve, buccal nerve, auriculotemporal nerve, lingual nerve, and inferior alveolar nerve) [3, 6] (Fig. 6).

### VI cranial nerve - Abducens nerve

The abducens nerve is a motor nerve that emerges from the abducens nucleus, located just beneath the floor of the IV ventricle in the dorsal pons [3]. The nerve courses anteriorly to exit the brainstem at the pontomedullary junction and crosses prepontine cistern in a dorsal-to-ventral direction. Successively, it descends near to the posterior aspect of the clivus, passing through the Dorello’s canal, and enters in the cavernous sinus [9]. It is the unique cranial nerve that courses through the central venous portion of the sinus. Finally, it enters the orbit via the superior orbital fissure to innervate the lateral rectus muscle [3, 6] (Fig. 5).

### VII cranial nerve - Facial nerve

**Fig. 7:**
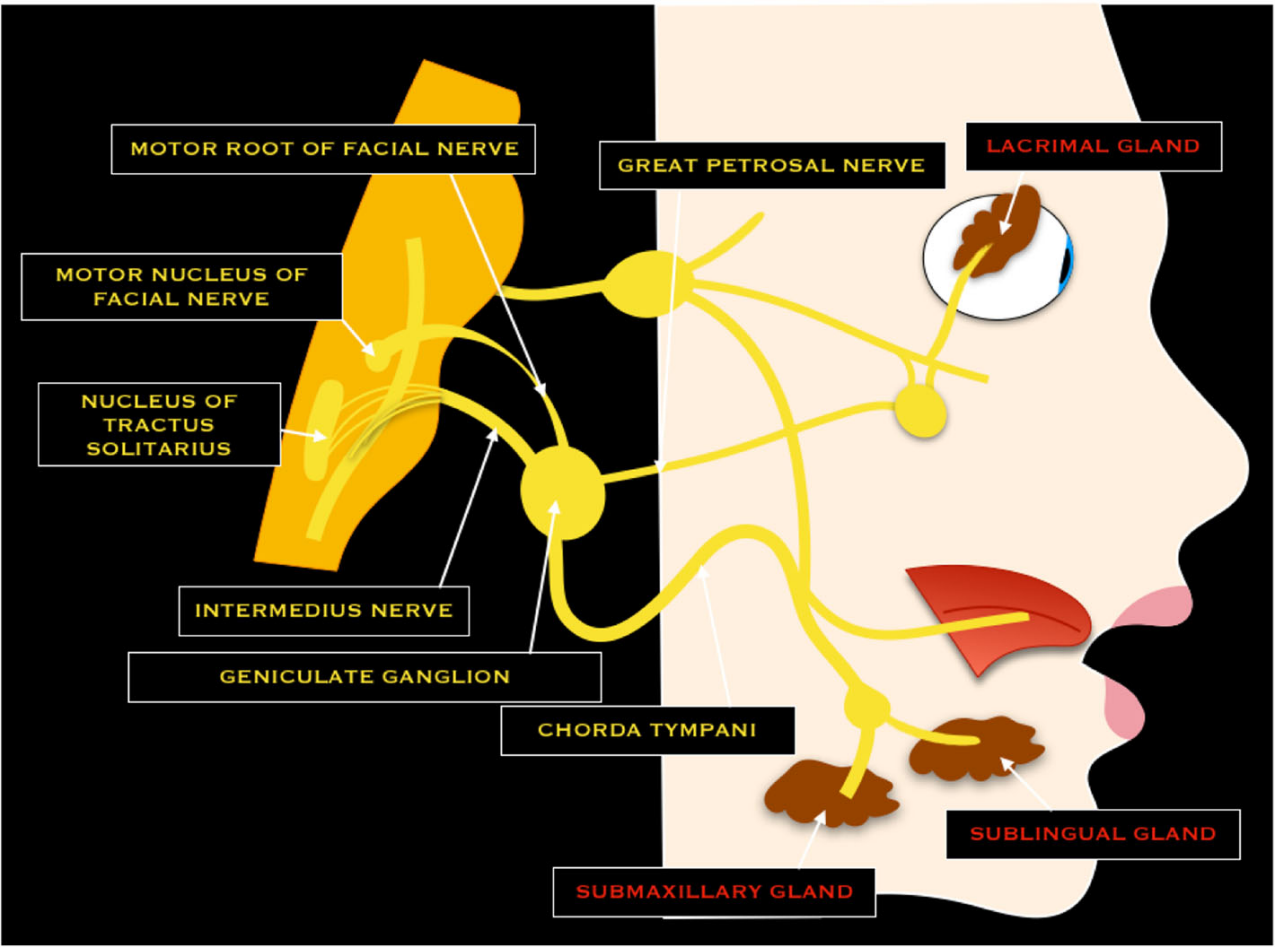
Schematic drawing of the facial nerve

The facial nerve is a mixed nerve and consists of two portions: the proper VII nerve (whit motor function) and the intermediate nerve (with sensory and parasympathetic motor fibers) [6]. The motor nucleus is located in the lower portion of the pons and motor fibers exit from the lateral portion of the pontomedullary sulcus. Successively, the nerve traverses the cerebellopontine cistern and enters the temporal bone through the internal acoustic meatus. In the petrous bone, it passes through the facial canal, giving three segments (labyrinthine, tympanic, and mastoid). The nerve exits the skull base at the stylomastoid foramen and reaches the parotid gland [6]. From the mastoid portion, the nerve of the stapedius muscle and corda tympani emerge [6]. The afferent sensory fibers that originate from receptors of the first two-thirds of the tongue arrive at the geniculate ganglion, via the chorda tympani. They run with the intermediate nerve in the internal acoustic meatus and the cerebellopontine cistern to terminate in the nucleus of tractus solitarius. The parasympathetic fibers of the intermediate nerve arise from the superior salivatory nucleus. At the geniculate ganglion, the great petrosal nerve provides parasympathetic fibers for the lacrimal gland and the mucosa of mouth, nose, and pharynx [6] (Fig. 7).

### VIII cranial nerve - Vestibulocochlear nerve

**Fig. 8:**
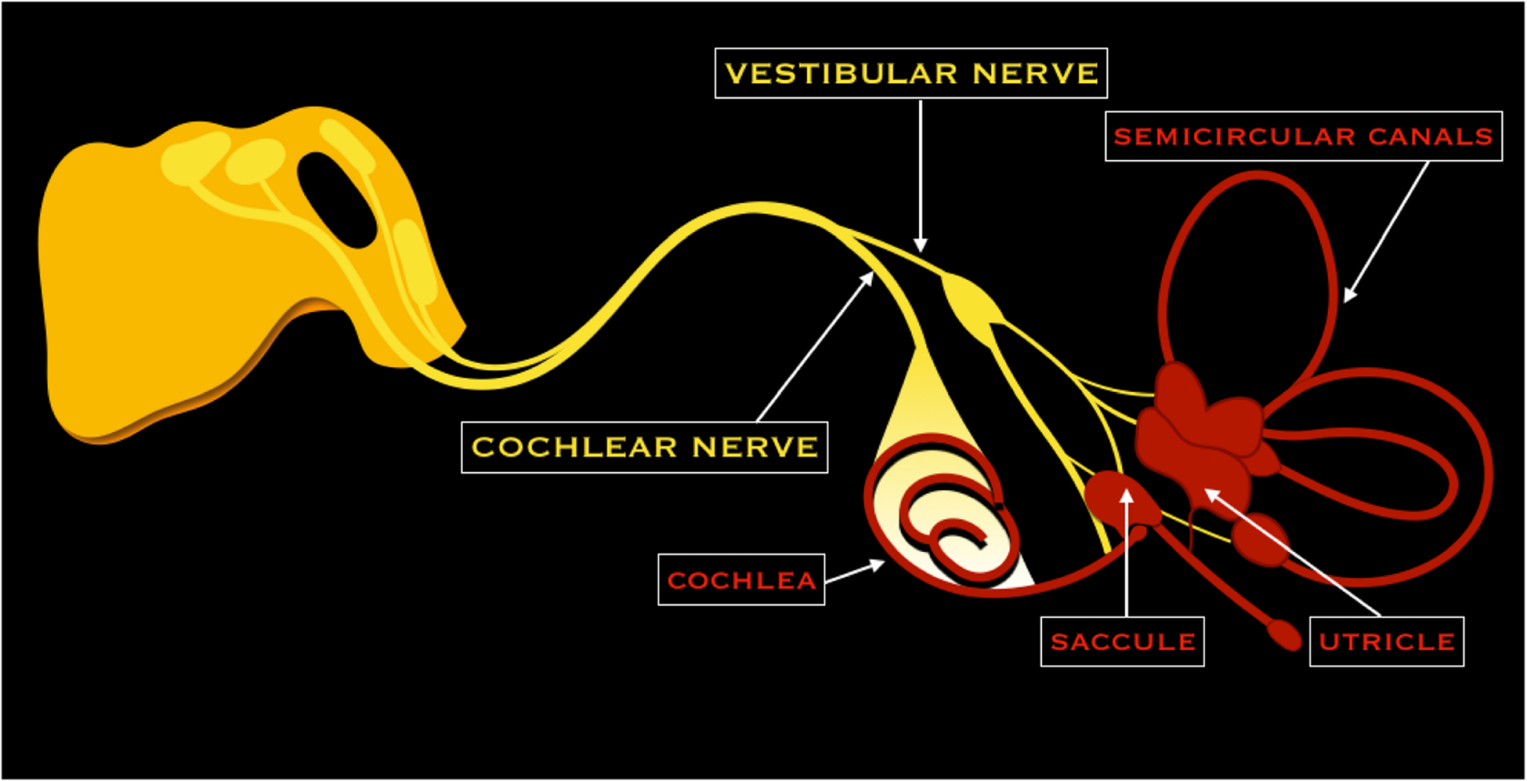
Schematic drawing of the vestibulocochlear nerve

The vestibulocochlear nerve is formed by two nerves: the cochlear nerve that transmits sound and originates in the cochlear membrane, and the vestibular nerve (divided in superior and inferior) that controls balance and originates from the ciliary sensory cells in the membranous labyrinth [10]. Both nerves traverse the internal acoustic meatus, together with the facial nerve, and pass in the cerebellopontine cistern. In the internal acoustic meatus, the nerve is so placed: the facial nerve is anterosuperior, the vestibular superior nerve is posterosuperior, the cochlear nerve is anteroinferior, and the inferior vestibular nerve is posteroinferior (for this, remember the acronym “Seven Up Coke Down”). Successively, vestibulocochlear nerve enters the brainstem and reaches the dorsal and ventral cochlear nucleus and the four vestibular nuclei that are located in the dorsolateral pons [6] (Fig. 8).

### IX cranial nerve - Glossopharyngeal nerve

**Fig. 9:**
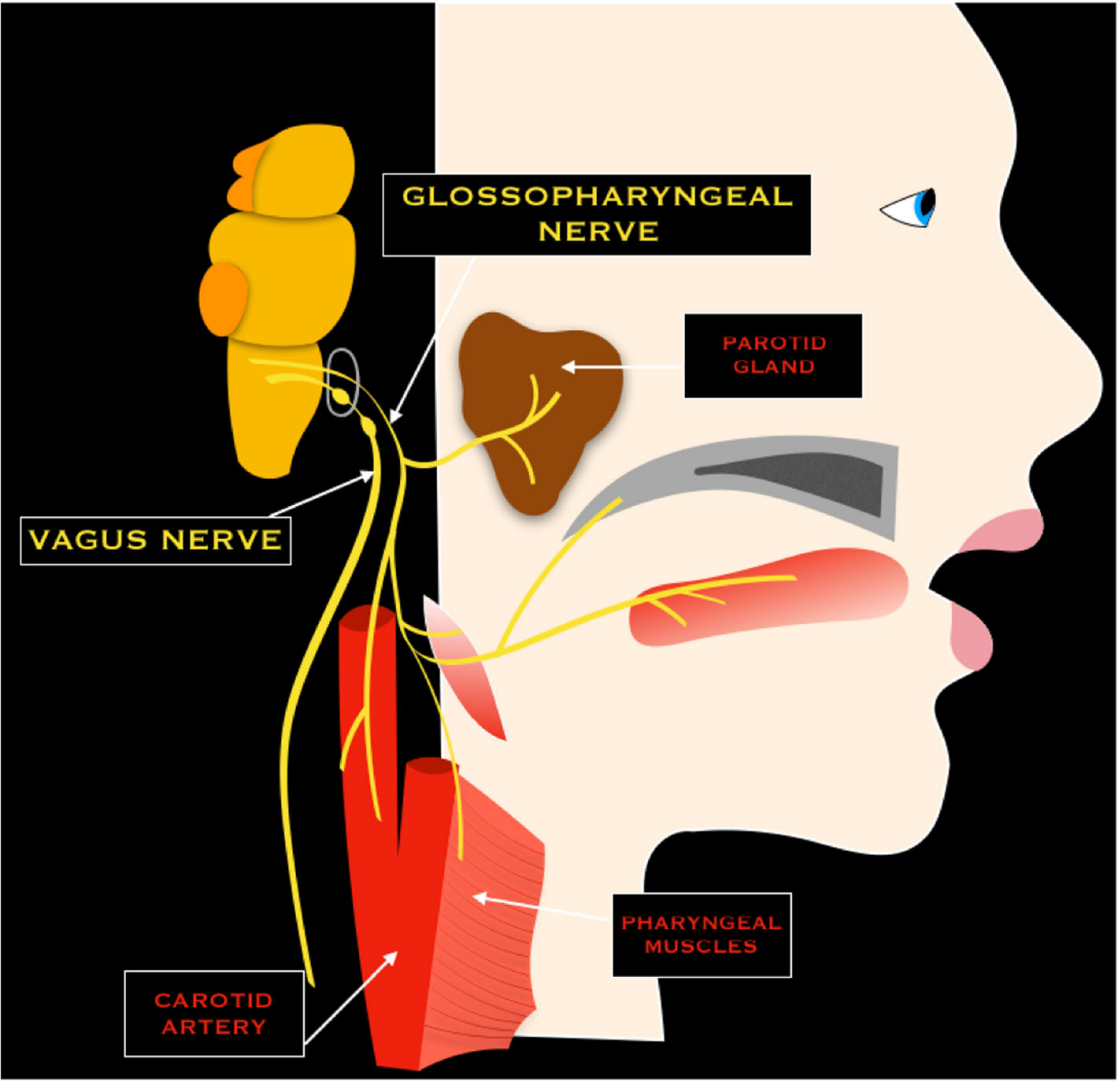
Schematic drawing of the glossopharyngeal and vagus nerves

The glossopharyngeal nerve is a mixed sensory, motor, and secretory nerve. It emerges from the lateral medulla into the lateral cerebellomedullary cistern, where it is closely associated with the flocculus of cerebellum [3]. Successively, the nerve exits the skull through the jugular foramen (pars nervosa), and it enters in the carotid space; successively, it turns lateral to the carotid artery and the stylopharyngeal muscle and continues lateral to the palatine tonsil area. Its lingual branch ends in the posterior sublingual space, to innervate the posterior third of the tongue (taste and sensory). Other nervous branches of the extracranial segment of IX nerve are pharyngeal (sensation from posterior oropharynx and soft palate), sinus nerve (parasympathetic supply to carotid body and sinus), stylopharyngeus (motor innervation to the stylopharyngeus muscle), and tympanic branch or Jacobson nerve (sensory information from the middle ear) [6] (Fig. 9).

### X cranial nerve - Vagus nerve

The vagus nerve emerges from the lateral medulla, from the base of the nucleus ambiguus and the dorsal nucleus of the vagus [6]. The nerve enters the lateral cerebellomedullary cistern and exits the skull through the jugular foramen (pars vascularis) between the glossopharyngeal nerve and the accessory nerve. Into and below the jugular foramen, the nerve forms the superior ganglion, and just below the jugular foramen, the nerve forms the inferior vagal ganglion. Successively, it descends vertically in the retrostyloid space together with the carotid artery, to join the upper mediastinum where it gives off the recurrent laryngeal nerve. Successively, the vagus nerve enters the abdominal cavity through the esophageal hiatus [6] (Fig. 9).

### XI cranial nerve - Accessory nerve

**Fig. 10:**
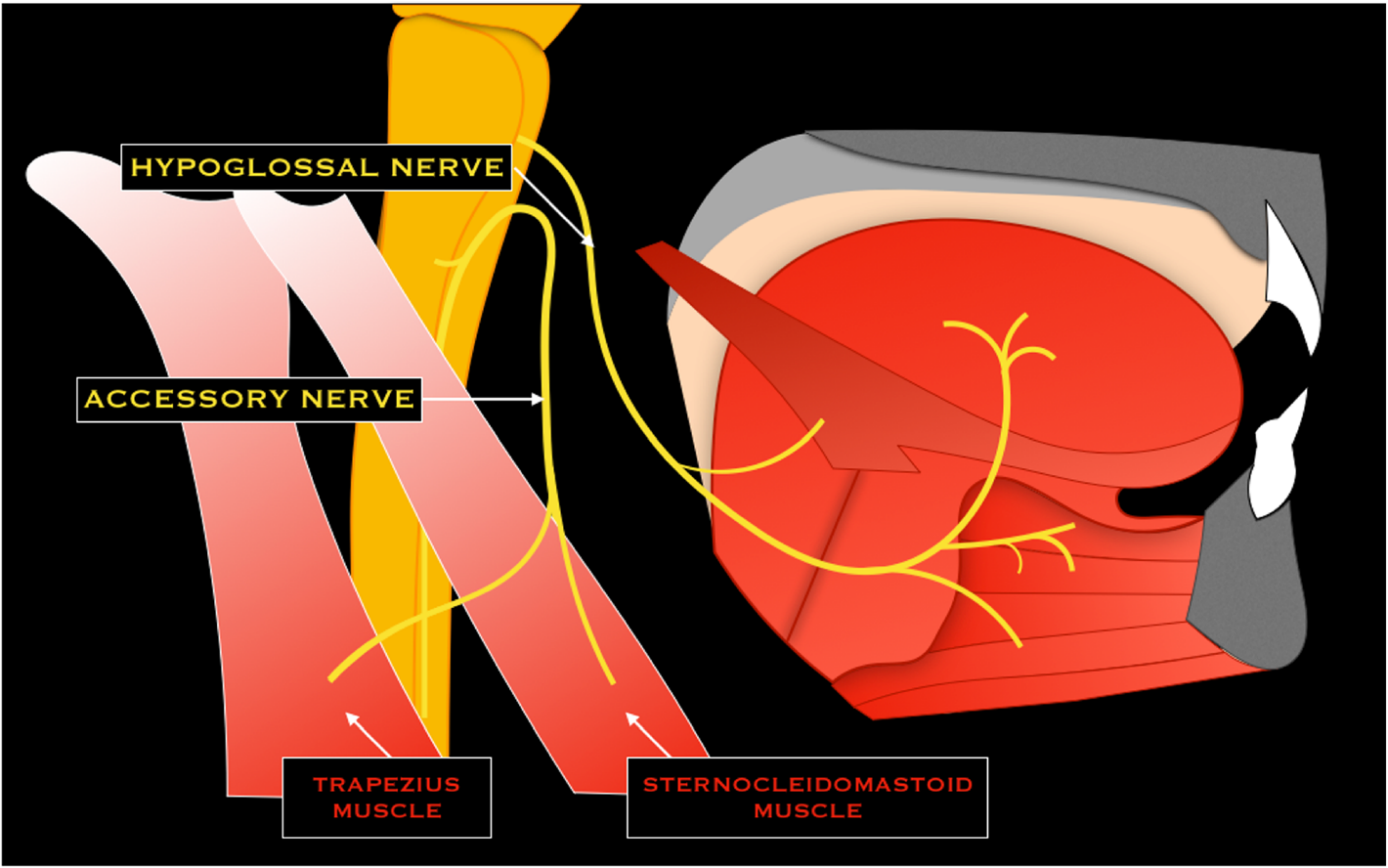
Schematic drawing of the accessory and hypoglossal nerves

The accessory nerve has both cranial and spinal roots [3]. Cranial roots emerge into the lateral cerebellomedullary cistern, while spinal roots emerge from upper cervical segment of the spinal cord (from C0 to C5) and pass superiorly through the foramen magnum into the cisterna magna. Successively, conjoined cranial and spinal roots enter the jugular foramen, posterior to the glossopharyngeal and vagus nerve. Within the jugular foramen, cranial roots mix with vagus nerve at the level of the superior vagal ganglion, while spinal roots descend laterally to the internal carotid artery and internal jugular vein, and achieve the sternocleidomastoid and trapezius muscle [3, 6] (Fig. 10).

### XII cranial nerve - Hypoglossal nerve

The hypoglossal nerve emerges from the medulla oblongata as a series of rootlets extending from the ventrolateral sulcus of the medulla into the lateral cerebellomedullary cistern, near to the vertebral artery and the posterior inferior cerebellar artery (PICA). The nerve exits the skull trough the hypoglossal canal and then runs medial to the glossopharyngeal, vagus, and accessory nerves, deeply to the digastric muscle, to innervate a large part of extrinsic and intrinsic tongue muscles [3] (Fig. 10).

## Pathology

Pathology of cranial nerves involves a wide spectrum of etiologies that includes primary or secondary neoplastic involvement, inflammation, infections, traumatic injuries, ischemic lesions, compression by adjacent structures, and congenital pathologies (Table 5).

**Table 5:** Summary of pathologies that may involve cranial nerves

Neoplasm	
▪ Primary neural tumor	
▪ Compressive mass (sellar, paracavernous, bone tumor)	
▪ Leptomeningeal or perineural spread of tumor	
Inflammation	
▪ Optic neuritis	
▪ Multiple sclerosis, neuromyelitis optica spectrum disorders	
▪ Pseudotumor (Tolosa-Hunt syndrome)	
▪ Sarcoidosis	
▪ Gradenigo syndrome	
▪ Vestibular neuritis, labyrinthitis	
Infection	
▪ Abscess, encephalitis, meningitis, thrombophlebitis, viral neuritis (Ramsey-Hunt syndrome)	
▪ Skull base osteomyelitis	
Trauma	
Vascular	
▪ Neurovascular compression, aneurysm	
Ischemia	
Hemorrhage	
▪ Superficial siderosis	
Congenital	
▪ Neurofibromatosis (I and II), Kallman syndrome, aplasia/hypoplasia of the optic nerve, Duane syndrome, Moebius syndrome	

Here, we review and illustrate the most important and frequent pathologies that may involve cranial nerves.

All images and cases were collected in our Institute.

## Neoplasms

### Schwannoma and neurofibroma

**Fig. 11:**
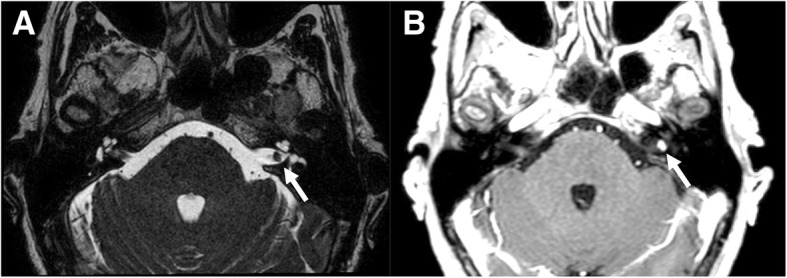
Small intracanalicular vestibulocochlear schwannoma. MRI steady-state free procession (SSFP) (**a**) and T1-weighted post-gadolinium (**b**) axial images show a small hypointense lesion on steady-state sequence with avid contrast-enhancement visible in the intracanalicular segment of internal auditory canal (arrows)

**Fig. 12:**
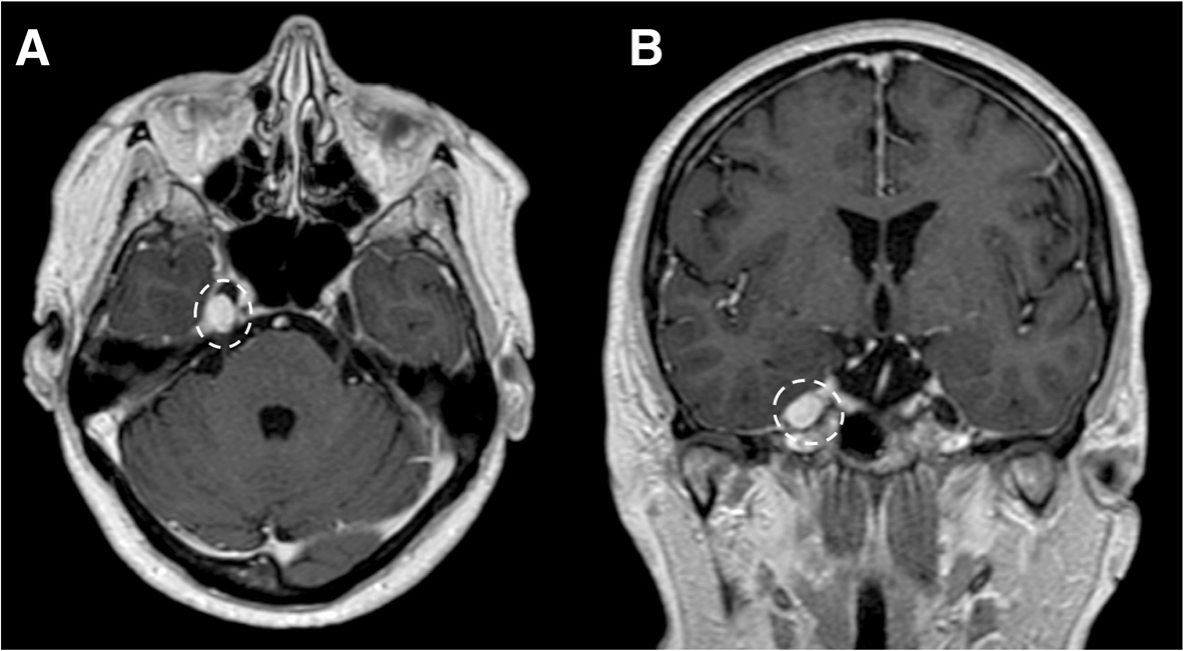
Small trigeminal schwannoma. MRI T1-weighted post-gadolinium axial (**a**) and coronal (**b**) images show a homogeneous enhancing mass in the right Meckel’s cave (dotted circles)

**Fig. 13:**
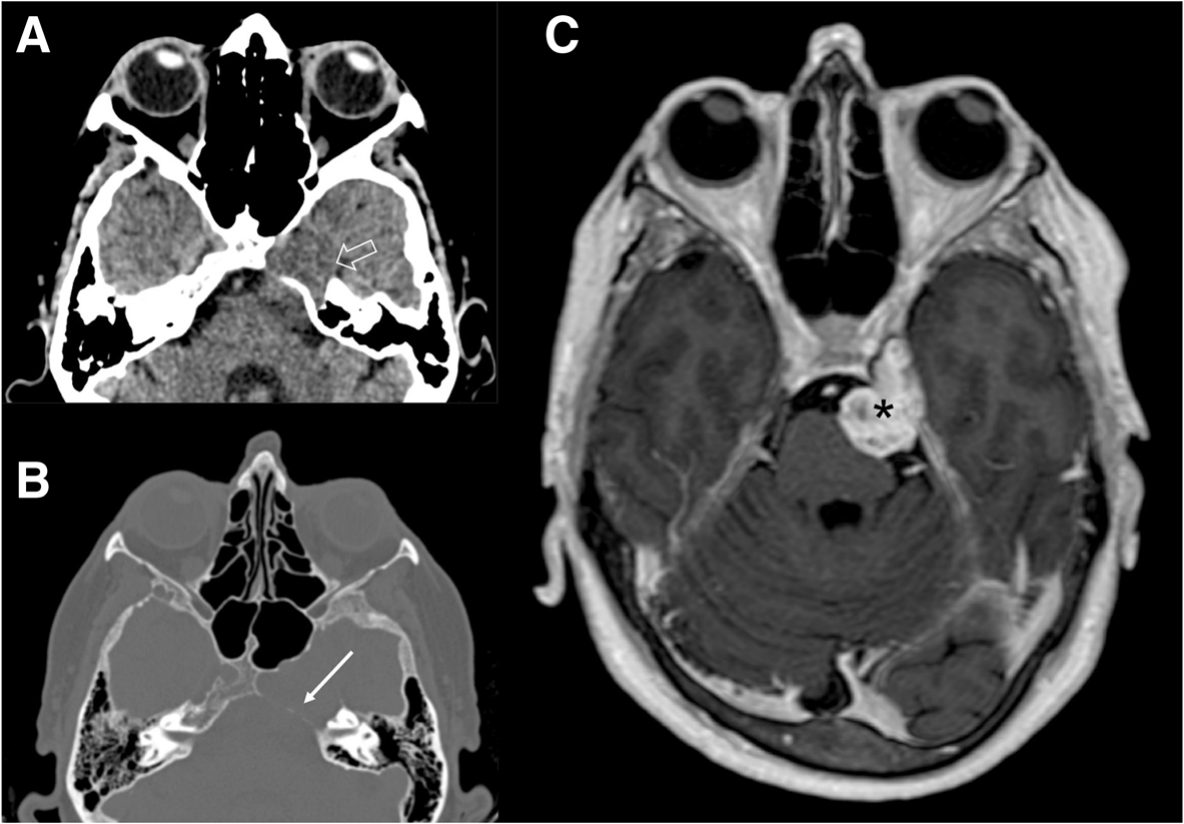
Large trigeminal schwannoma. CT axial image (soft tissues and bone windows, **a**, **b**, respectively) demonstrates a mild iso-attenuated mass (empty arrow) with erosion of the left petrous apex (arrow); MRI T1-weighted post-gadolinium axial image (**c**) shows an avid enhancing dumbbell-shaped mass (asterisk), that extends in the Meckel’s cave, along the cisternal course of the left trigeminal nerve

**Fig. 14:**
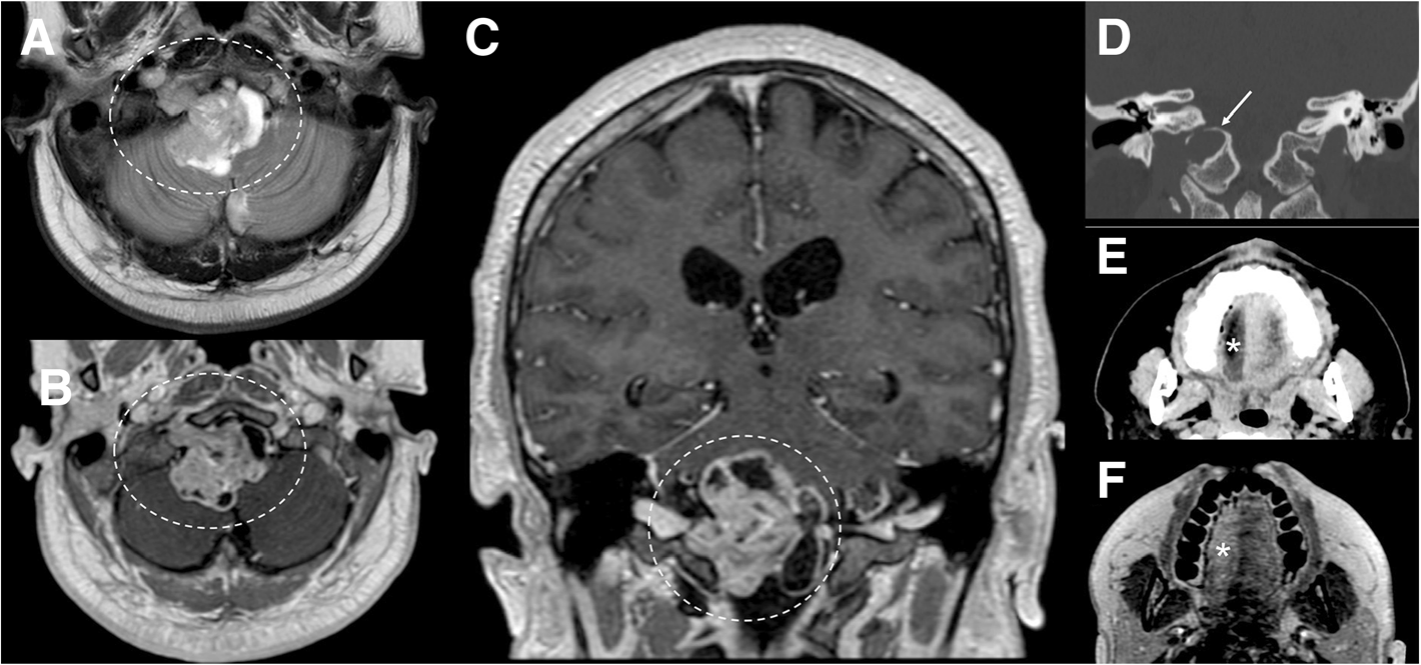
Hypoglossal schwannoma. MRI T2-weighted axial image (**a**), T1-weighted post-gadolinium axial (**b**), and coronal images (**c**) demonstrate an extra-axial expansive lesion (dotted circles), mildly hyperintense with heterogenous contrast enhancement, surrounded by cystic components. The mass is located along the course of the right hypoglossal nerve. CT (bone window, **d**) clearly shows the enlargement of the right hypoglossal canal (arrow). Atrophy of the right tongue muscles (asterisks) is well visible at CT (**e**) and T1-weighted axial sequence (**f**) as hypodense and hyperintense area, respectively

Schwannoma is a benign nerve sheath tumor that originates from Schwann cells. Approximately 90% of intracranial schwannomas arise from the VIII cranial nerve with trigeminal involvement as the second most common (0.8–5%) [11, 12]. Intracranial schwannomas can be associated with neurofibromatosis 2 (NF2), a genetic condition characterized by the presence of multiple schwannomas, meningiomas, and ependymomas. Patient with NF2 may have bilateral vestibular schwannomas or non-vestibular schwannomas [13]. Imaging shows the slow growth of the tumor, with bone remodeling of neural foramen and deformation of the adjacent brain tissue. Features of end-organ compromise can be present. On CT, schwannomas have low-intermediate attenuation with variable enhancement. MR imaging findings include iso-hypointensity on T1-weighted sequences, high signal on T2-weighted sequences, and marked enhancement on post-contrast images, eventually with unenhanced cystic spaces [13] (Figs. 11, 12, 13, and 14).

**Fig. 15:**
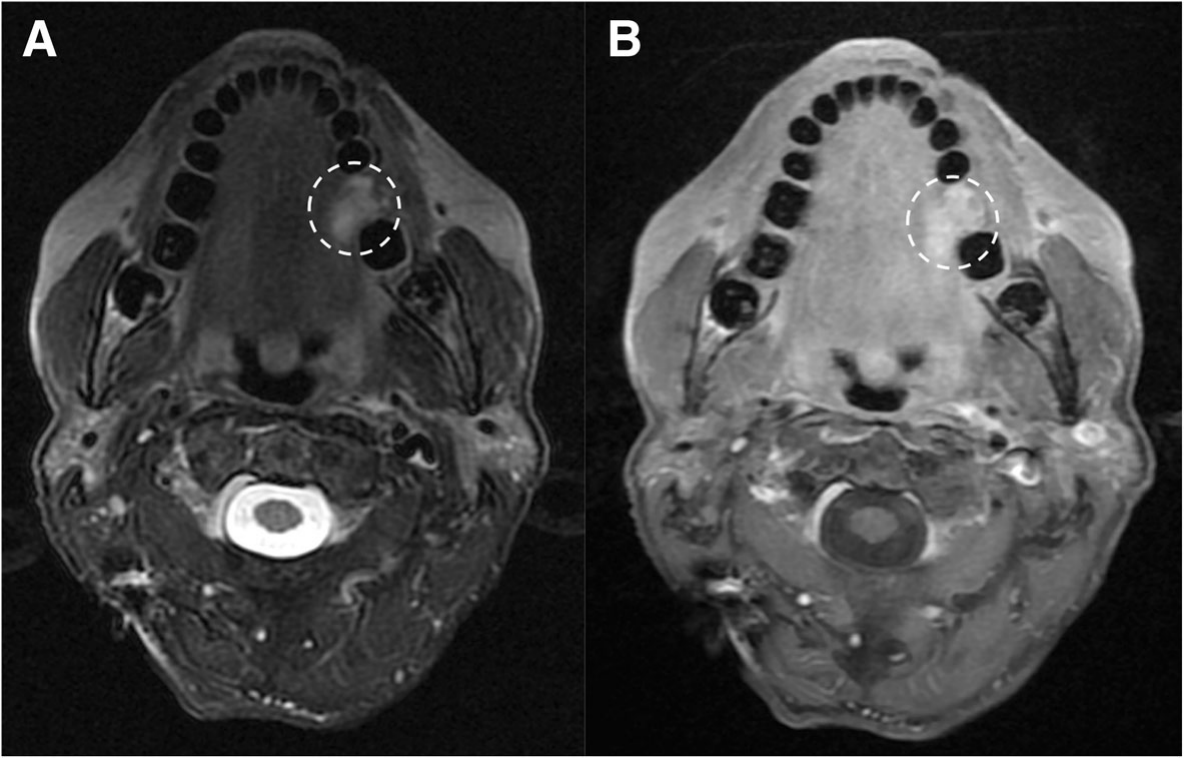
Neurofibroma of the lingual nerve. Patient with NF1. MRI T2-weighted fat-suppressed (**a**) and T1-weighted post-gadolinium (**b**) axial images, display an hyperintense and enhanced round mass along the course of the left lingual nerve (dotted circles)

**Fig. 16:**
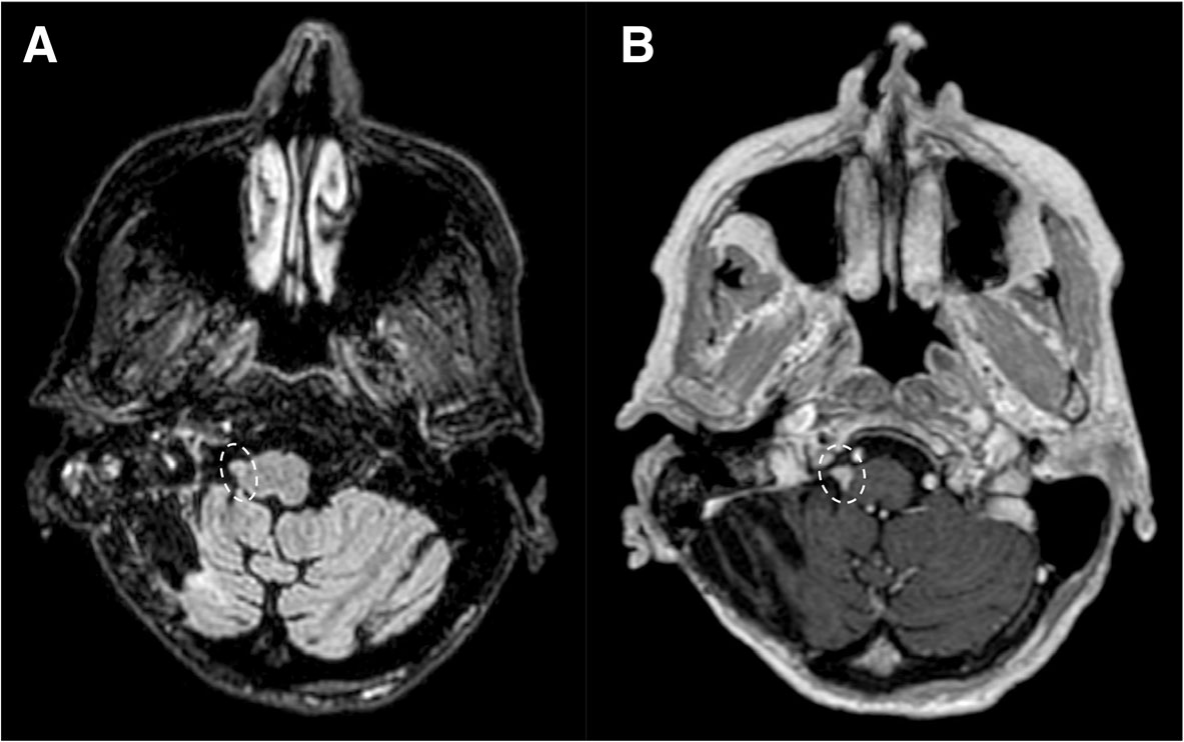
Small glossopharyngeal neurofibroma. Patient with NF1. MRI FLAIR (**a**) and T1 post-gadolinium (**b**) sequences, axial plane, demonstrate a mildly hyperintense small lesion (dotted circles) with avid enhancement located on the right post-olivary sulcus

Neurofibromas rarely involve cranial nerves, and are more commonly found extracranially. They are typically associated with neurofibromatosis 1 (NF1) and can show malignant transformation. NF1 is a genetic condition characterized by the presence of *café au lait spots*, neurofibromas, optic nerve glioma, osseous lesions, iris hamartomas, and axillary or inguinal freckling (Figs. 15 and 16).

### Esthesioneuroblastoma

**Fig. 17:**
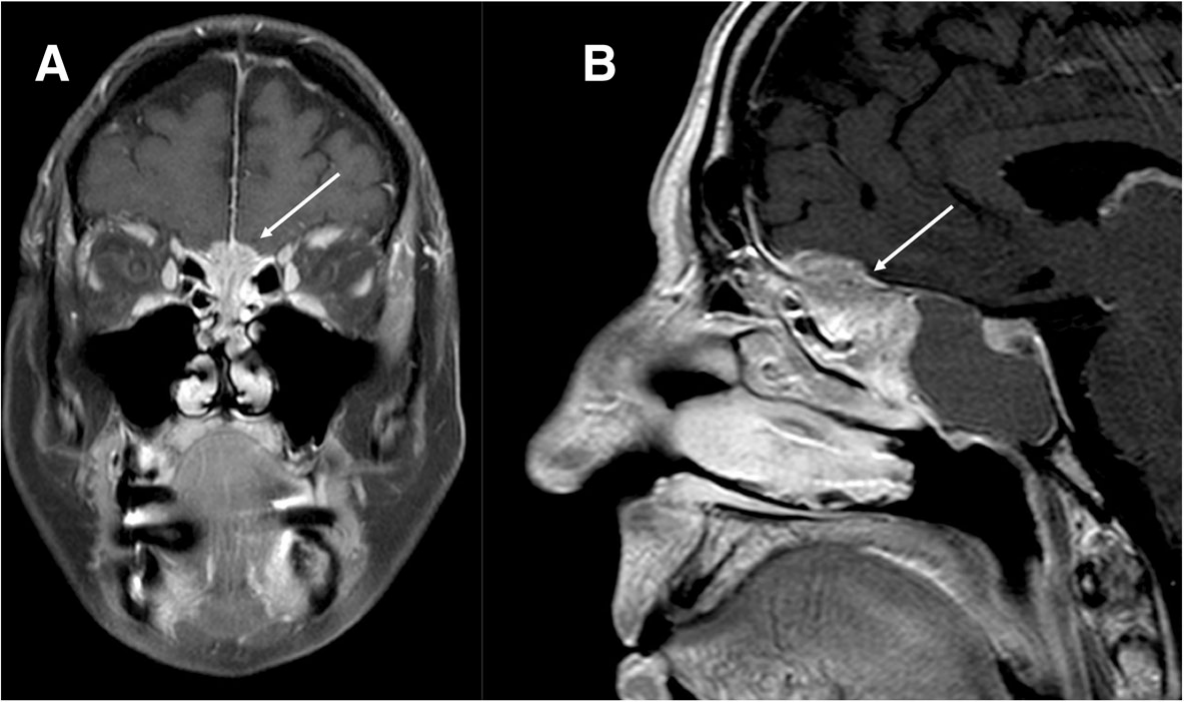
Esthesioneuroblastoma. MRI T1-weighted fat-suppressed post-gadolinium sequence, coronal (**a**) and sagittal (**b**) images show a marked enhanced large median mass (arrows) in the sinonasal region growing superiorly into the inferior frontal region of the brain

Esthesioneuroblastoma (ENB) or olfactory neuroblastoma is a rare malignant tumor of neural crest origin, arising from the olfactory epithelium of the nasal vault. It presents typically in the second and sixth decades of life, without gender predilection [14]. Clinical presentation may include, in a various combination, epistaxis, nasal obstruction, decreased olfactory function, diplopia, and proptosis. ENB may invade paranasal sinuses, orbits, and anterior cranial fossa, and, if metastatic, involve local lymph nodes, with distant metastasis to lungs, liver, and bone. At CT, ENB does not have specific features appearing as a homogeneous soft tissue mass in the nasal cavity. However, CT is useful to evaluate bone involvement. On MRI, ENB usually appears hypointense on T1-weighted sequences and intermediate to hyperintense on T2-weighted sequences. Contrast enhancement is avid and homogeneous, except for areas of necrosis or hemorrhage. MRI may be useful to detect dural involvement [14] (Fig. 17).

### Optic nerve glioma

**Fig. 18:**
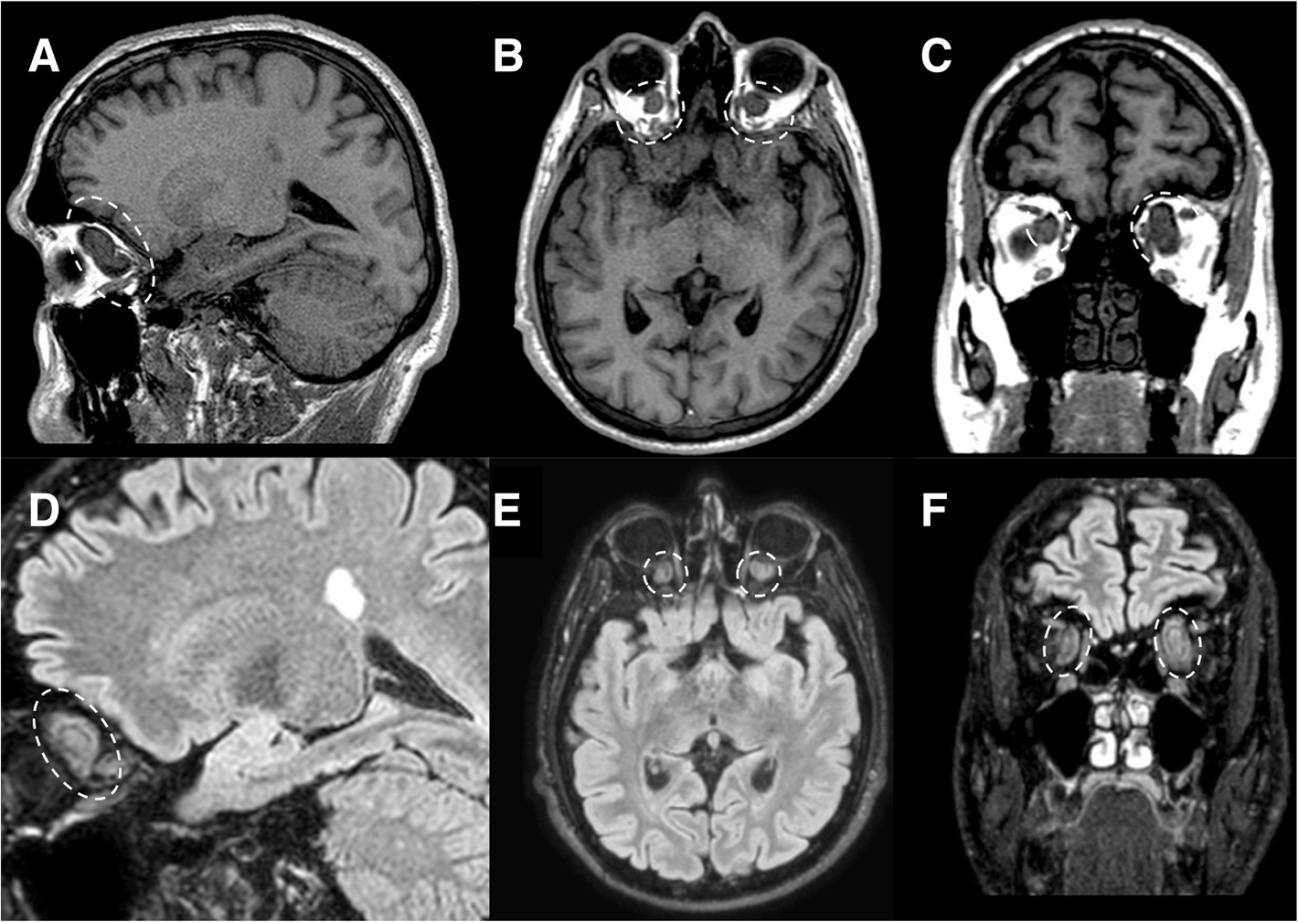
Optic nerve gliomas. MRI T1-weighted sagittal (**a**), axial (**b**), and coronal (**c**) images show bilateral intraorbital hypointense masses (dotted circles) centered on the optic nerve. FLAIR images (**d**–**f**) well depict the isointense masses causing enlargement of the optic nerves (dotted circles)

Optic nerve glioma is a relatively rare tumor that typically occurs in children. If associated with NF1, glioma can be multifocal and bilateral. In NF1 patient, optic nerve is the most common site of the tumor, whereas in non-NF1 patients, chiasma involvement is the most frequent. On MRI, the tumor typically appears isointense on T1-weighted images and iso-hyperintense on T2-weighted images, with variable contrast enhancement; in non-NF1 patients, cystic components may be detected (Fig. 18). If present, perineural arachnoidal gliomatosis shows hyperintense signal on T2-weighted sequences and may have contrast enhancement [8].

### Perineural spread of tumor

**Fig. 19:**
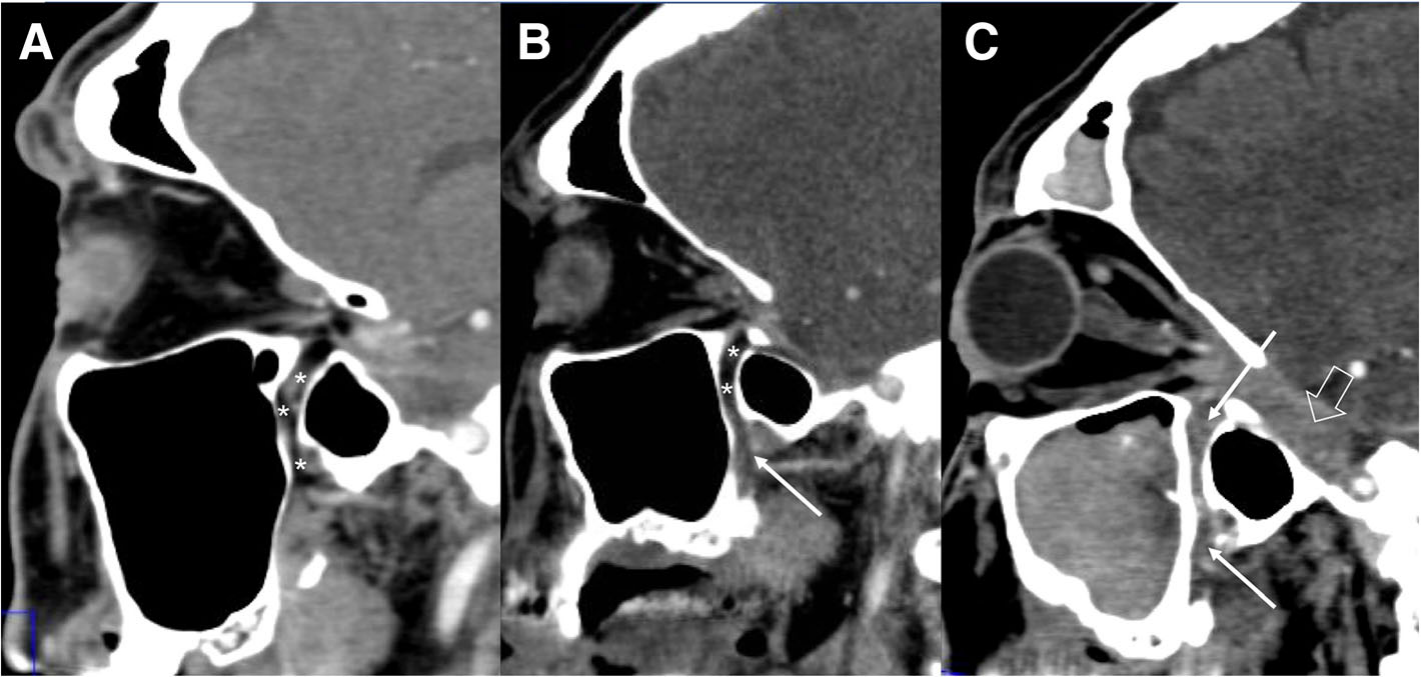
Perineural spread of tumor. Patient with squamous carcinoma of the retromolar trigone. CT sagittal images at 0 (**a**), 6 (**b**), and 12 (**c**) months follow-up. Note the progressive infiltration (arrows) of the pterygopalatine fossa (asterisks) with obliteration of the normal fatty tissue. In (**c**), it is evident the invasion of Meckel’s cave (empty arrow)

**Fig. 20:**
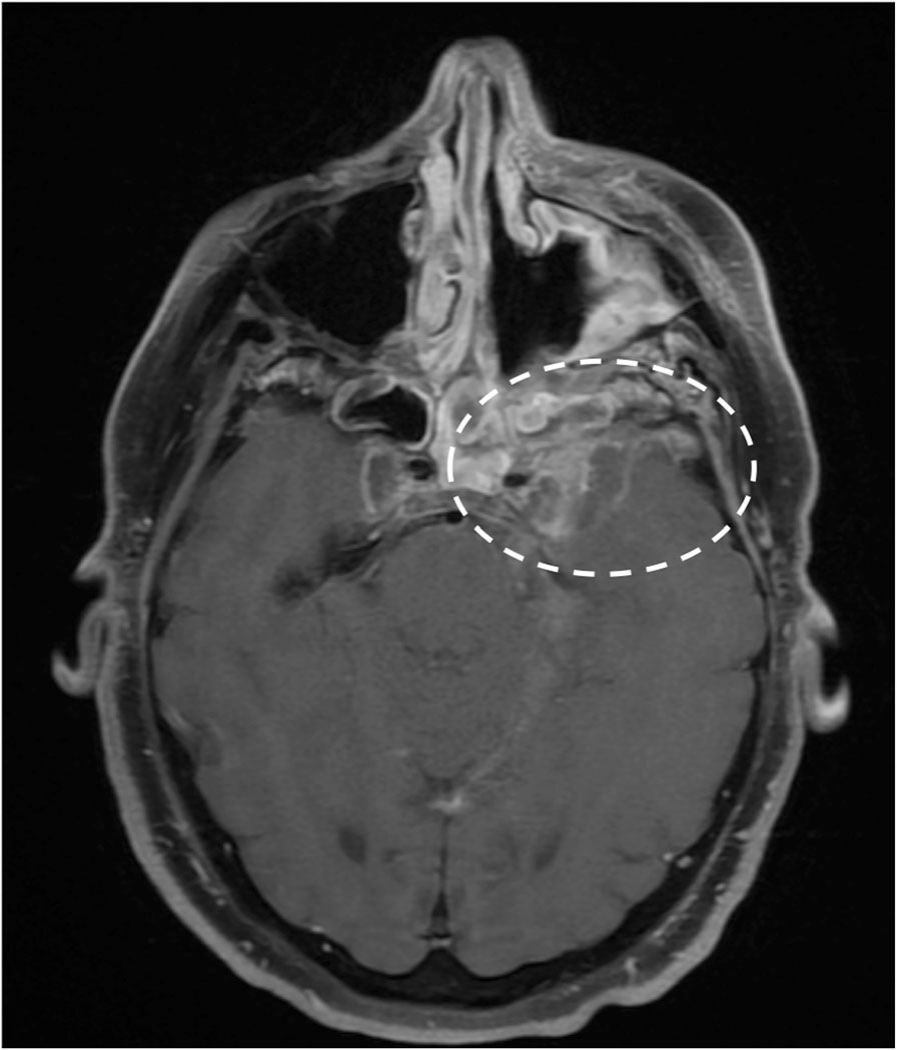
Perineural spread of tumor. Patient with squamous carcinoma of the retromolar trigone. MRI T1-weighted post-gadolinium image shows diffuse neoplastic enhancement in the left pterygopalatine fossa, cavernous sinus and Meckel’s cave (dotted circle)

**Fig. 21:**
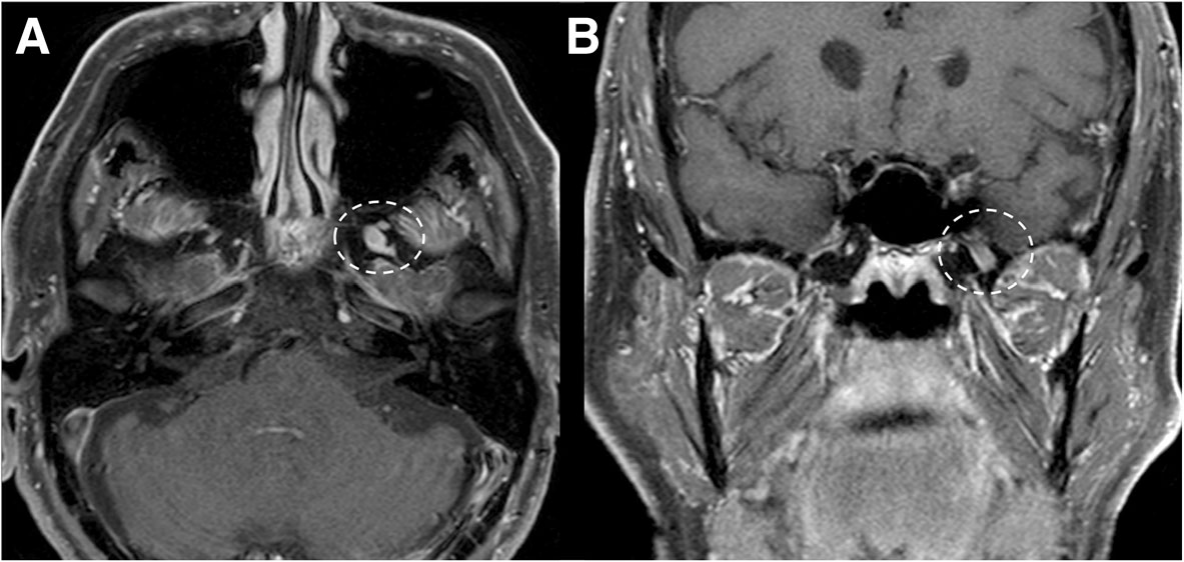
Perineural spread of tumor. MRI T1-weighted post-gadolinium axial (**a**) and coronal (**b**) images. Note the enlargement and the enhancement of the mandibular branch of trigeminal nerve (V2) (dotted circles)

Two types of perineural growth of tumor are described: perineural invasion (PNI), a microscopical process that affect small nerves, and perineural spread of tumor (PNS) that extends along the sheaths covering larger central nerves [15]. The incidence of perineural spread is about 2.5–5% and may occur with any head and neck malignancy [16, 17]. It is typically associated with carcinoma arising from minor or major salivary glands (e.g., adenoid cystic carcinoma), mucosal or cutaneous squamous cell carcinoma, basal cell carcinoma, melanoma, lymphoma, and sarcoma. In most cases, the peripheral branches of second (V2) and third (V3) divisions of the trigeminal nerve, and the descending branch of the facial nerve serve as conduit for perineural spread. Also, the ophthalmic nerve and the hypoglossal nerve may be interested [17]. Clinical findings include neural dysfunction symptoms, as pain, dysesthesias, or muscle denervation atrophy [5]. For radiologist, the knowledge of the anatomy of cranial nerves is crucial to evaluate the perineural spread. On MRI, it can be visualized as segmental nerve thickening and enhancement on post-gadolinium sequences. Typically, imaging techniques with fat suppression are used to better increase the conspicuity of the enhancement [17]. Skull base invasion may result as replacement of normal fatty marrow signal [5] (Figs. 19, 20, and 21). Tumoral growth may cause enlargement of foramina or canals through which nerves exit the skull.

### Leptomeningeal spread of tumor

**Fig. 22:**
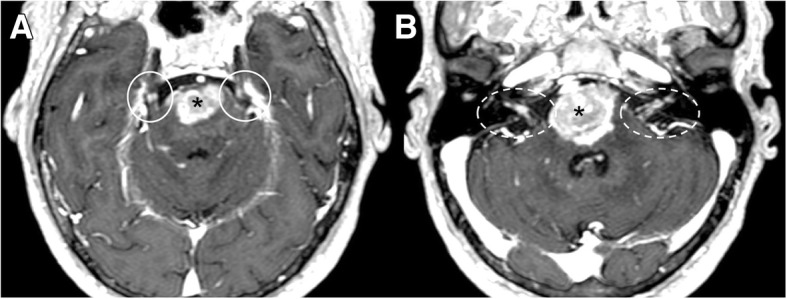
Leptomeningeal spread of tumor. Patient with lung carcinoma. MRI axial image of T1-weighted post-gadolinium sequence demonstrates a large metastasis of the pons (asterisks), bilateral leptomeningeal enhancement of trigeminal nerves (**a**, circles), and bilateral facial and vestibulocochlear nerves (**b**, dotted circles)

Primary (medulloblastoma, ependymoma, oligodendroglioma, and glioblastoma) and secondary (leukemia/lymphoma, breast cancer, lung cancer, and melanoma) tumors may spread through subarachnoid spaces with extension along the cranial nerves. VII and VIII cranial seem to be the most affected nerves [18]. Clinically, symptoms of irritation and neural compression are present with multiple cranial neuropathies and mental status changes, secondary to meningeal irritation and hydrocephalus. The nerve may enhance on post-gadolinium sequences and appear enlarged (Fig. 22). Neural enhancement can be very subtle and better demonstrated on post-Gadolinium fluid-attenuated inversion recovery (FLAIR) sequences [5, 19–21].

### Meningioma

**Fig. 23:**
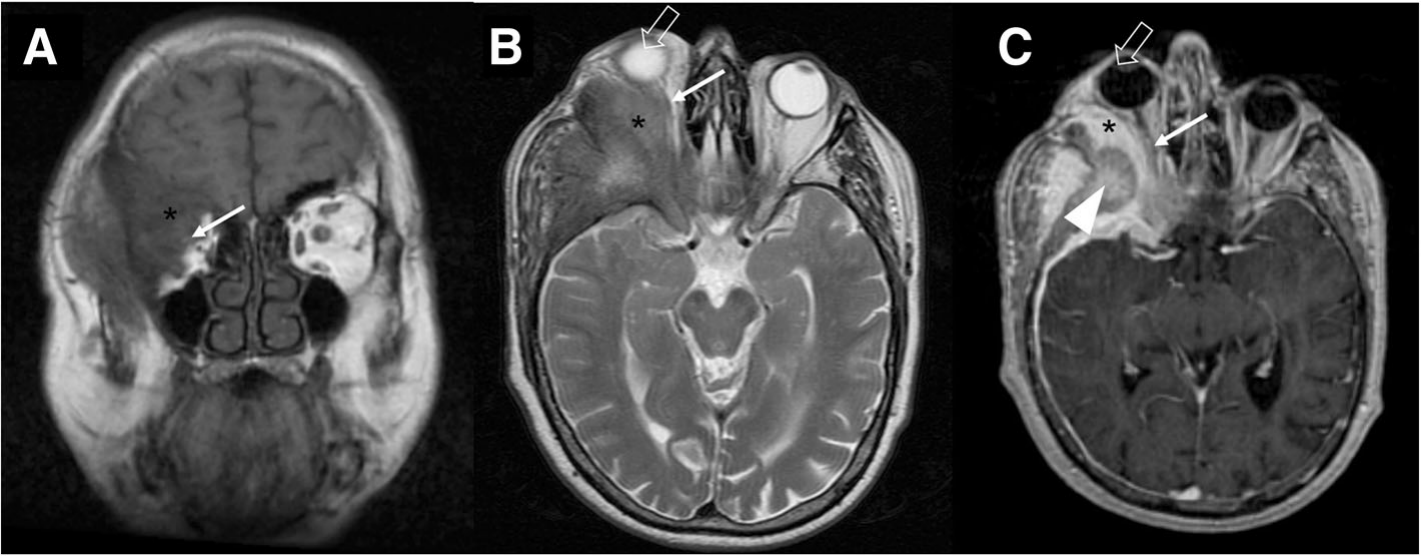
Sphenoid wing meningioma. MRI T1-weighted coronal image (**a**), T2-weighted (**b**), and T1-post gadolinium (**c**) axial images. A large mass with avid enhancement is visible, suggestive for meningioma of the right sphenoid wing (asterisks) with orbital involvement, displacement of the optic nerve (arrows), and the globe (empty arrows). Signs of hyperostosis are also evident (arrowhead)

Meningioma is the commonest extra-axial tumor of central nervous system and arises from the meningothelial cells of arachnoid layer. Typical locations include parasagittal and lateral aspect of the cerebral convexity, sphenoid wing, middle cranial fossa, and olfactory groove. The tumor may show transforaminal extension, for example into the orbit or following the trigeminal course. After the acoustic schwannoma, it represents the second most common mass in the cerebellopontine angle. On imaging, meningioma appears as a lobular, extra-axial mass with well-circumscribed margins and a broad-based dural attachment. On MRI, it is isointense/hypointense relative to gray matter on the T1-weighted sequences and isointense/hyperintense relative to gray matter on the T2-weighted sequences. A rim of high T2 signal that separates the mass from the adjacent brain is often visible (“cleft signs”). Post-contrast sequences show marked and homogeneous enhancement with rare central nonehancing areas, corresponding to necrosis or calcification phenomena. In up to 72% of cases, a “dural tail” is evident, as enhancement of the neighboring dura mater. Possibly associated bony changes can be osteolysis, hyperostosis, and enlargement of the adjacent foramina [21] (Fig. 23). Meningioma may also affect the optic nerve, appearing as homogeneous enhancing lesion around the optic nerve, with intermediate T1 and T2 signal [22].

## Inflammation

### Optic neuritis

**Fig. 24:**
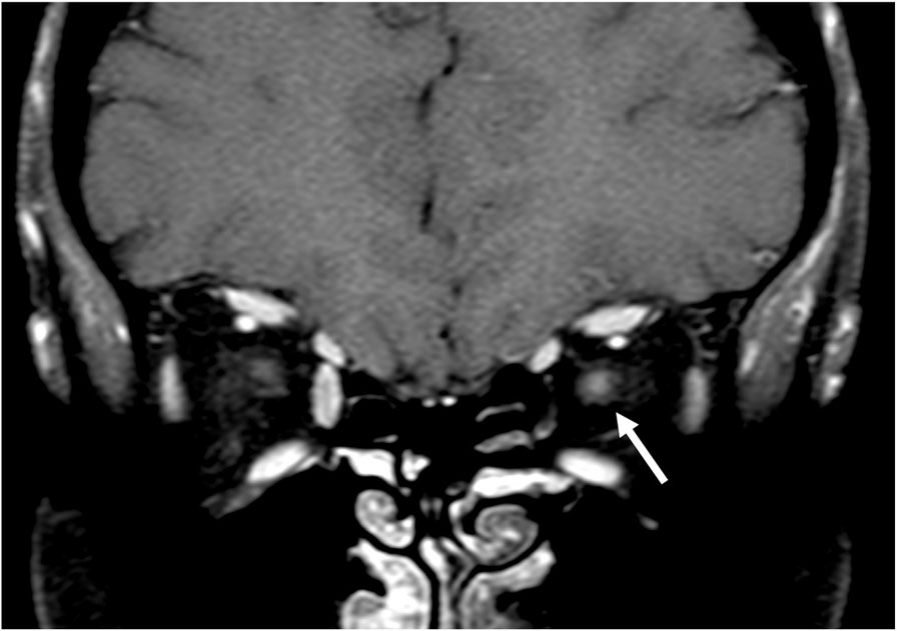
Optic neuritis. MRI T1-weighted post gadolinium fat-saturated coronal image. Note the enlargement and the enhancement of the left optic nerve (arrow)

The term optic neuritis (ON) usually refers to inflammatory process that involve optic nerve. Clinically, it presents with painful eye movements and visual loss. On MRI, the nerve appears swollen and hyperintense on T2-weighted sequences, with contrast enhancement best seen on fat-suppressed T1-weighted sequences [8] (Fig. 24). ON may be observed in autoimmune (for example multiple sclerosis and neuromyelitis optica spectrum disorders) and systemic diseases (sarcoidosis, lupus erythematosus, Wegener disease, Sicca syndrome,  Behcet disease) [23]. Particularly, in neuromyelitis optica spectrum disorder (NMOSD), nerve involvement may be bilateral and more severe than in multiple sclerosis (MS). NMOSD is also characterized by involvement of spinal cord, with brain MRI features not suggestive for MS [8].

### Tolosa-Hunt syndrome

**Fig. 25:**
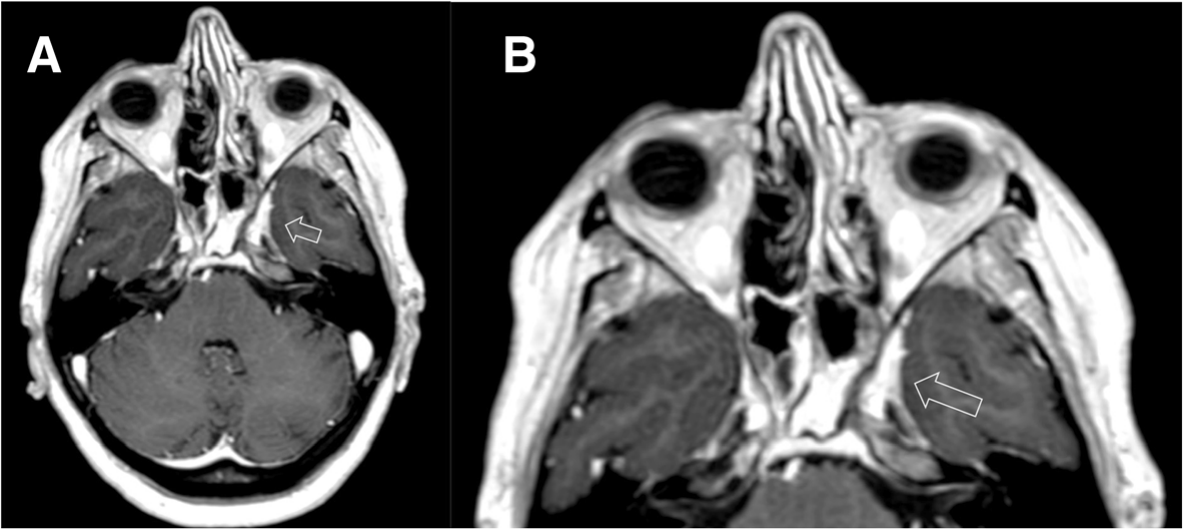
Tolosa-Hunt syndrome. MRI T1-weighted post-gadolinium axial image (**a**, and enlargement in **b**) shows enhancing inflammatory infiltration in the left orbital apex extending into the cavernous sinus (empty arrows)

Tolosa-Hunt is an uncommon disease related to a retro-orbital pseudotumor extending in the cavernous sinus [24, 25]. It is an idiopathic disorder that can manifest with a unilateral painful ophthalmoplegia, diplopia, and deficit of the V1 branch of trigeminal nerve. Bilateral pseudotumor is described in 5% of cases [24]. Tolosa-Hunt consists in a granulomatous inflammation of the lateral wall of the cavernous sinus or superior orbital fissure. Imaging techniques can show an infiltrative soft tissue mass within the cavernous sinus that appears enlarged. The tissue is hypo-isointense on T2-weighted sequences, with avid contrast enhancement on post-gadolinium images (Fig. 25). Differential diagnosis may include lymphoma, sarcoidosis, metastatic disease, meningioma, infection, granulomatous pachimeningitis, Meckel’s cave schwannoma, aneurysm, and pituitary macroadenoma [25]. Clinical findings may overestimate this condition and MRI is critical to exclude other similar conditions, allowing for precise management and therapeutic planning. After diagnosis, a good response to steroid therapy can be achieved.

### Bell’s palsy

**Fig. 26:**
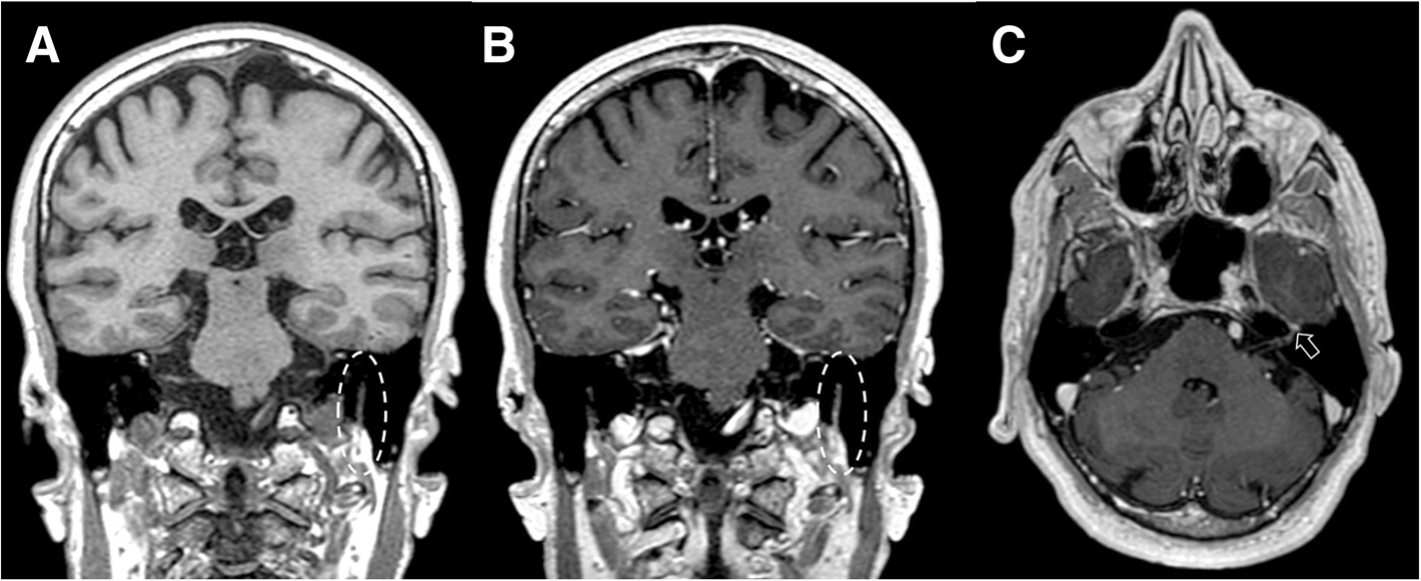
Bell’s Palsy. MRI T1-weighted pre- (**a**) and post-gadolinium (**b**), coronal images; T1-weighted post-contrast axial image (**c**). In **a**, **b**, note the swelling and enhancement of the left facial nerve (dotted circles); in **c**, the enhancement around the geniculate ganglion (arrow)

Bell’s palsy (idiopathic facial palsy) is the most common cause of unilateral peripheral nerve neuropathy. Clinical presentation is variable, with dysgeusia, mastoid pain, impaired salivation, and lacrimation. A *Herpes-Simplex-Virus* reactivation seems to be the most probable etiology [5]. Prognosis is often good and the treatment is based on corticosteroid therapy, often with the addition of acyclovir. Typical Bell’s palsy does not require imaging studies. However, MRI may be useful to exclude other lesions responsible of atypical Bell’s palsy (gradual-onset palsy, failure to improve with time) or recurrent palsy [26]. On MRI, the facial nerve shows increased enhancement on post-contrast sequences that may involve one or more segments, without nodularity (Fig. 26). Enhancement of distal intrameatal and labyrinthine segments is typical [26]. On T2-weighted sequences, the nerve may be hyperintense. In case of irregular or nodular enhancement, other causes of pathology (such as perineural spread of tumor) should be evaluated. Bilateral Bell’s palsy is unusual, often associated with HIV or other systemic pathologies like sarcoidosis, Lyme, and syphilis [5, 26–28].

### Vestibular neuritis and labyrinthitis

**Fig. 27:**
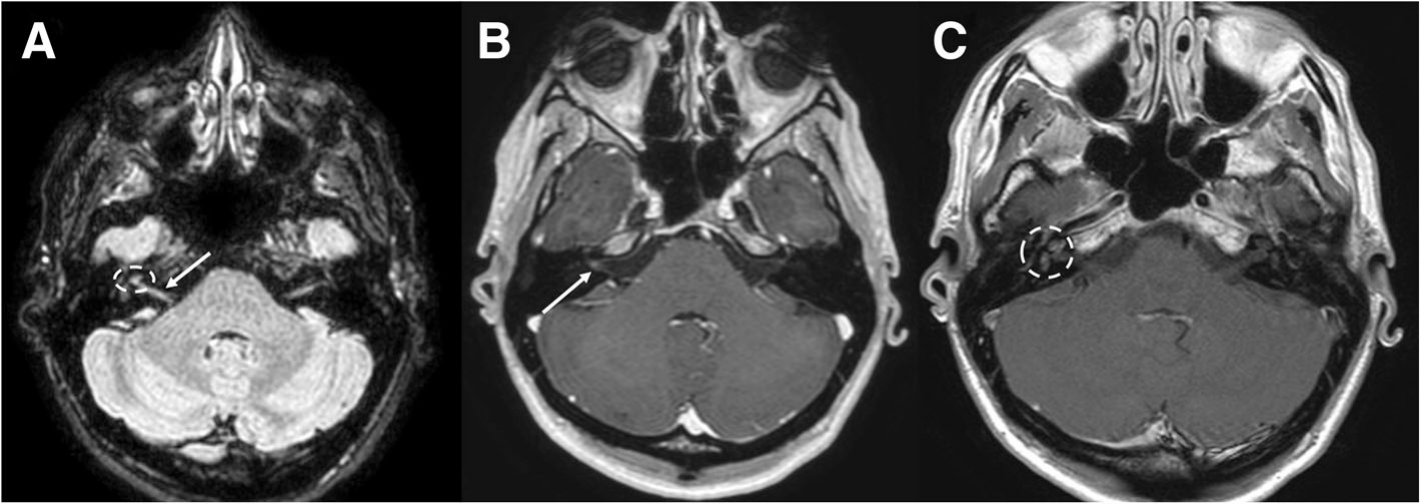
Vestibular neuritis. MRI FLAIR (**a**) and T1-weighted post-gadolinium (**b**, **c**) axial images show mild hyperintensity (**a**, arrow) and enhancement (**b**, arrow) of the right vestibular nerve and right labyrinth (**a**, **c**, dotted circles)

Vestibular neuritis presents as unilateral acute vertigo without hearing loss. Nausea and vomiting are also associated. It affects young and mid-adults with no sexual preponderance. The etiology is various. Viral, bacterial, and protozoan infections have been reported, but also allergic and autoimmune causes are described. Inflammation of the vestibular nerve may be complicated by demyelination, with loss of function, not always reversible [29]. MRI shows hyperintensity of the cisternal tract of the vestibular nerve on T2-weighted and FLAIR sequences [30] with enhancement on post-gadolinium images [31] (Fig. 27). Acute labyrinthitis is similar to vestibular neuritis with associated hearing loss and tinnitus [5].

## Infection

**Fig. 28:**
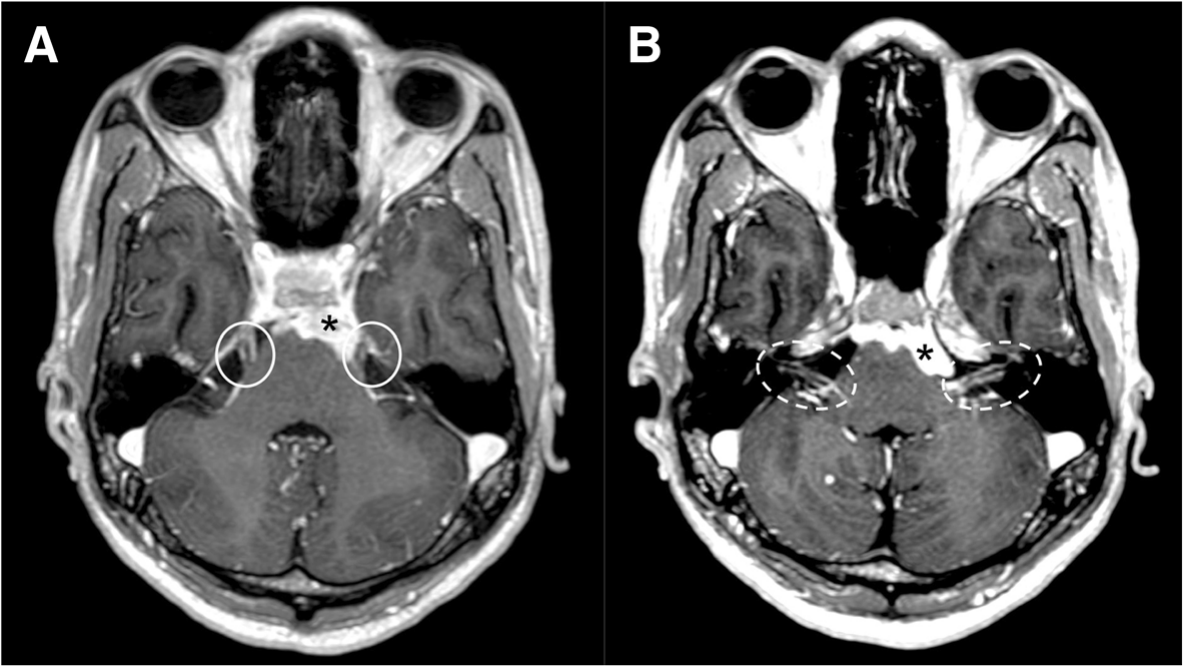
Tuberculosis (TBC). MRI T1-weighted post-gadolinium axial image demonstrates pathologic enhancing tissue involving the basal cistern and ventral surface of the brainstem (asterisks), with leptomeningeal enhancement of bilateral trigeminal (**a**, circles), facial, and vestibulocochlear nerves (**b**, dotted circles)

Infectious meningitis may result from viral, bacterial, fungal, or parasitic infection. Viral infections, mostly related to *Herpes simplex virus type 1*, C*ytomegalovirus*, and *Varicella zoster*, may manifest with cranial nerve involvement and abnormal enhancement of the nerve on MRI [28]. Bacterial meningitis is typically caused by *Haemophilus Influenzae*, *Streptococcus pneumoniae*, and *Neisseria Meningitidis*. Imaging is often normal, although MRI post-contrast sequences can demonstrate leptomeningeal enhancement [5]. Intracranial Tuberculosis, typically in pediatric population, can manifest as leptomeningitis with involvement of cranial nerves (Fig. 28). *Cryptococcus neoformans* is associated with the Cryptococcal meningitis, characterized by optic neuropathy. Necrosis of the optic nerve and chiasm by cryptococcal organism have been described. *Rhinocerebral mucormycosis* is fungal infection in immunocompromised patients, with sinonasal disease that may progress to the orbit and cavernous sinus. It can be complicated by vascular and perineural invasion and local thrombotic infarction. Cranial neuroschistosomiasis, less common than the spinal form, is characterized by a granulomatous reaction that leads to increase of intracranial pressure and focal neurologic signs. Lyme disease, caused by *Borrelia burgdorferi*, may involve any of the cranial nerves, with a predilection for the facial nerve, that appears thickened and enhanced [28].

## Trauma

**Fig. 29:**
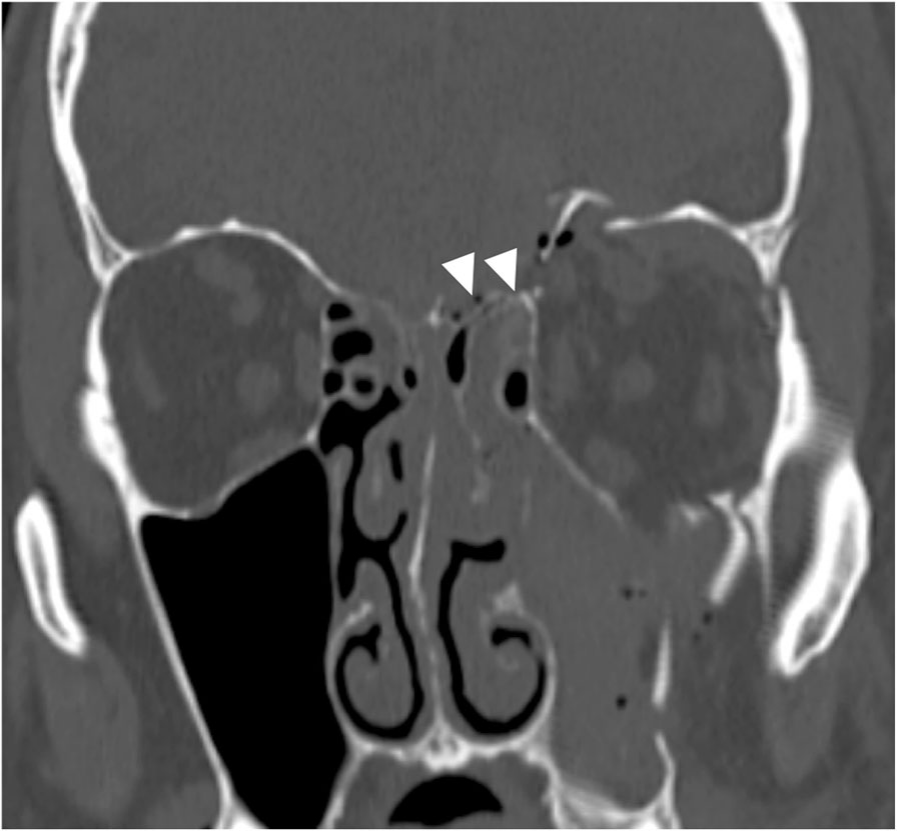
Fracture of cribriform plate. CT coronal image shows multiple fractures of the skull base. Note in particular the fracture of the cribriform plate (arrowheads)

Accidental or iatrogenic trauma, causing edema, hematoma, or disruption of the fibers, may lead to nerve impairment. Traction, stretching, impingement, and transection of the fibers are the typical mechanisms of injury [32]. High-resolution CT is the imaging modality of choice to detect skull base fractures and foraminal involvement. MRI may be useful to demonstrate intraneural edema or hemorrhage, especially on T2*-weighted sequences. Olfactory nerve may be involved in the closed injury of basal frontal lobe, with or without association of cribriform plate fracture (Fig. 29). Optic and oculomotor nerves may be affected in the orbital and optic canal fractures. Lesions of the abducens nerve are reported in the fracture of the clivus and petrous apex. Injuries of the facial nerve are associated to temporal bone fractures, that are classified as transverse or longitudinal based on their relationship to the long axis of the petrous temporal bone. They occur in 7% of temporal bone fractures with predilection for the geniculate ganglion area and the stylomastoid canal with edema, hematoma, or partial/complete transection of the nerve [5, 6, 33, 34]. Iatrogenic injuries may complicate a variety of surgical procedures. Facial nerve paralysis may follow temporal bone and parotid surgery, while lower cranial nerves may be involved during neck dissection; in particular, the recurrent laryngeal nerve may be injured during thyroid and parathyroid surgery [32].

## Vascular

### Neurovascular compression

**Fig. 30:**
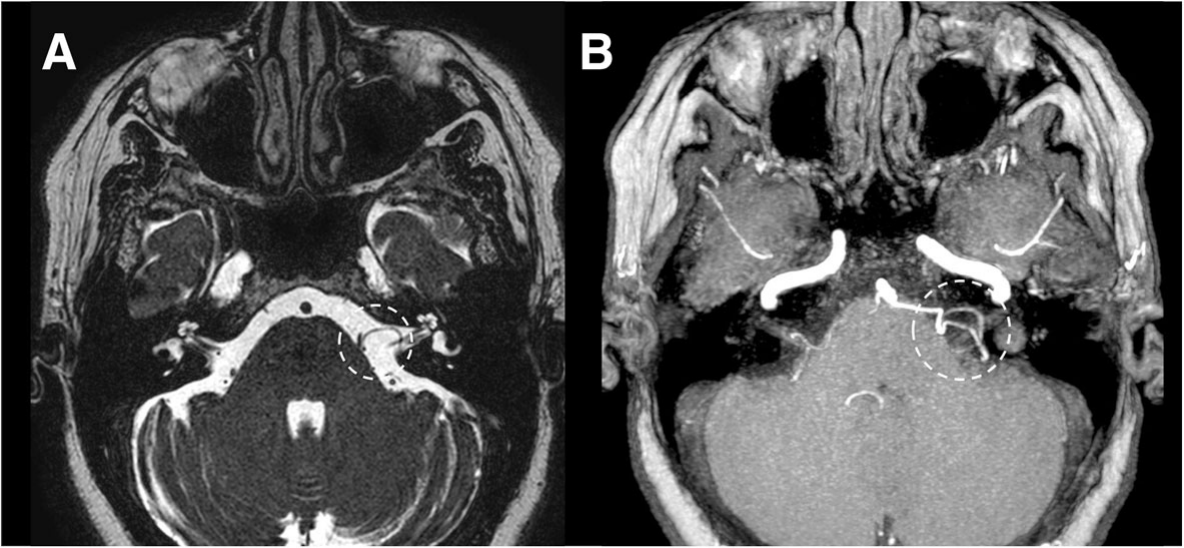
Neurovascular compression of facial nerve. Patient with left facial nerve palsy. MRI axial steady-state free procession (**a**) and T1-weighted post-gadolinium, MIP reconstruction (**b**) images show a neurovascular compression of the left facial nerve (dotted circles)

Neurovascular compression syndromes are caused by vascular structures (usually arteries) that directly contact the cisternal segment of a cranial nerve (Fig. 30). Typically, the transition zone between the central and the peripheral myelin is the most vulnerable region of the nerve [35]. Clinical presentation is variable and includes trigeminal and glossopharyngeal neuralgia, vestibular paroxysm, and hemifacial spasm. However, neurovascular compression should be evaluated only in the presence of specific clinical features [6]. Also, nonhemorrhagic aneurysms may be associated to nerve dysfunction, for example in the case of vision loss due to compression of II nerve or optic chiasm by proximal internal carotid artery, and III, IV, or VI cranial nerves’ palsy secondary to intracavernous aneurysm. Imaging may be useful to detect the displacement and the compression of a nerve by redundant arteries. Steady-state sequences and corresponding Time-of-Flight (TOF) or 3D T1-weighted gadolinium-enhanced images are very useful for diagnosis and preoperative evaluation [35].

## Ischemia

**Fig. 31:**
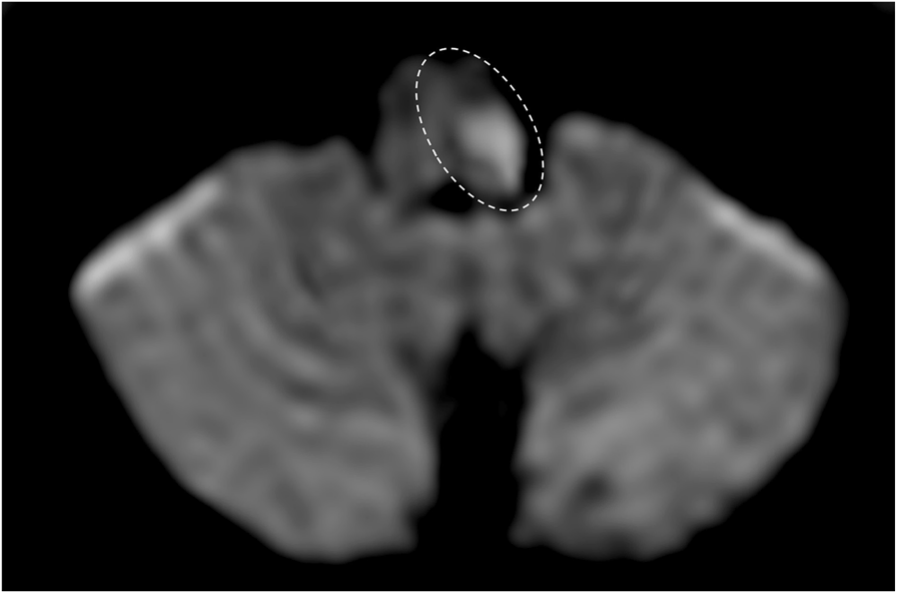
Wallenberg syndrome. Patient with left sensory loss, gait ataxia, nausea, and vertigo. MRI DWI (b1000) image demonstrates area of diffusion restricted in the left dorsal medulla oblongata, suggestive for recent ischemic lesion (dotted circle)

**Fig. 32:**
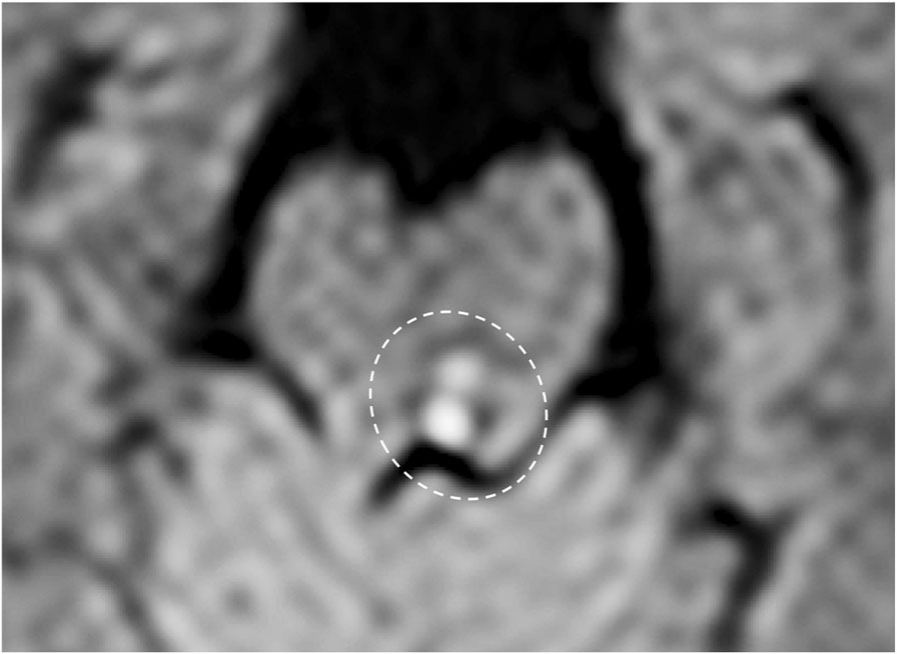
Trochlear nucleus ischemia. Patient with diplopia. MRI DWI (b1000) image shows restriction of the diffusion in the left posterior midbrain, in correspondence of the trochlear nucleus, suggestive for recent ischemic lesion (dotted circle)

Ischemic lesions of the brainstem may cause isolated nerve palsies, more commonly in elderly population. Cranial nerves  between the III and the VI are the most affected. Diffusion-weighted imaging (DWI) sequences are the best to visualize an acute ischemic lesion (Figs. 31 and 32), whereas FLAIR and T2-weighted sequences are useful to visualize subacute or old infarctions [6]. The anatomical knowledge of the nuclei of the cranial nerves is fundamental to interpret ischemic nerve palsies.

## Hemorrhage

### Superficial siderosis

**Fig. 33:**
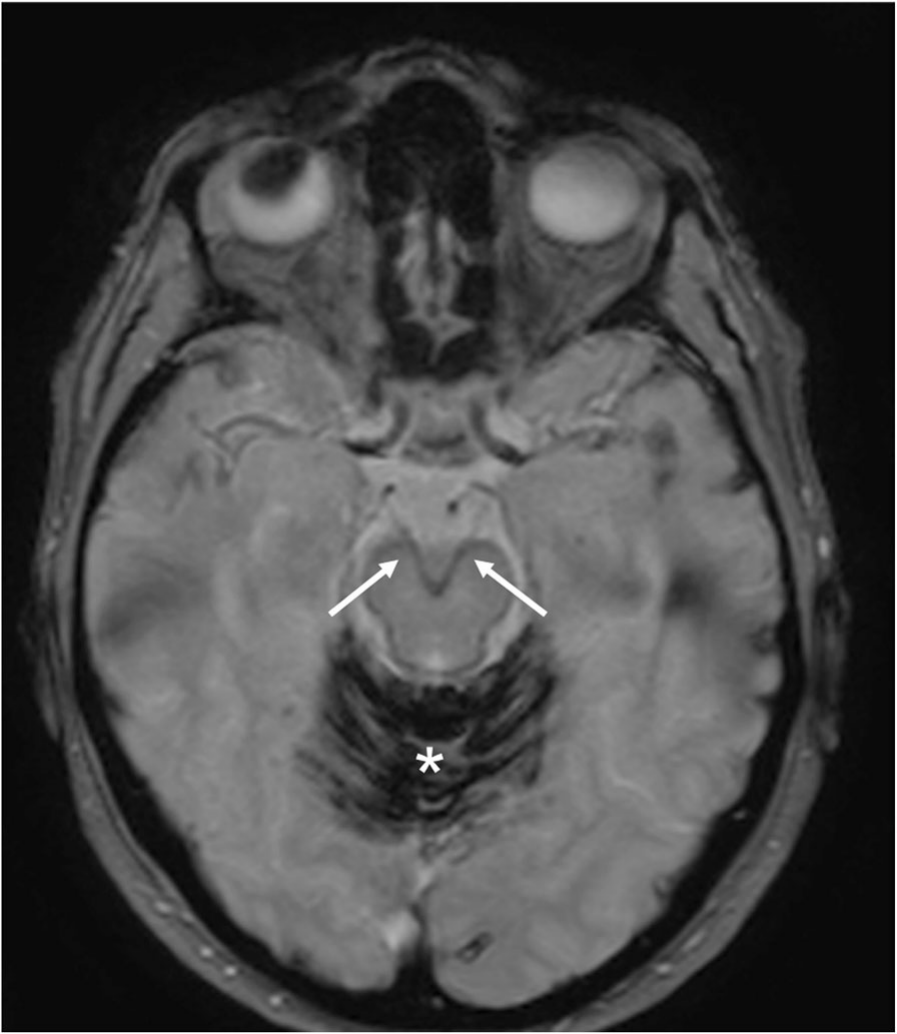
Superficial siderosis. MRI T2*-weighted axial image demonstrates hemosiderin deposition along the course of oculomotor nerves (arrows). Massive hemosiderin deposition is also visible on the cerebellar folia (asterisk)

Superficial siderosis is a rare pathological condition that results from chronic deposition of hemosiderin in the subpial layers of the brain and spinal cord. Classically, it is bilateral and it may present with the typical triad: ataxia, sensorineural hearing loss, and myelopathy. Other clinical presentations include dysarthria, nystagmus, myelopathy, bladder dysfunction, sensory and pyramidal signs. Hemosiderin deposition has predilection for the superior cerebellar vermis, cerebellar folia, frontal lobe, temporal cortex, brainstem, spinal cord, nerve roots and cranial nerve, mainly the VIII. Rarely, extraocular nerve palsies, optic and trigeminal neuropathy have been reported in literature. MRI is useful to identify hemosiderin deposition, based on magnetic susceptibility, better demonstrated as hypointensity on T2*-weighted sequences (blooming effect) (Fig. 33) [36].

## Conclusion

Cranial nerve dysfunctions may be the result of pathological processes of the cranial nerve itself or associated with tumor conditions, inflammation, infectious processes, or traumatic injuries. Neuroradiologists play a fundamental role in diagnosis. The knowledge of the anatomy of each nerve is crucial to detect the site of pathological alterations. Furthermore, it is necessary to know the most frequent pathologies of cranial nerves and their typical imaging features.

## Abbreviations

CSF: Cerebrospinal fluid
CT: Computed tomography
DWI: Diffusion-weighted imaging
ENB: Esthesioneuroblastoma
FLAIR: Fluid-attenuated inversion recovery
MRI: Magnetic resonance imaging
MS: Multiple sclerosis
NF1: Neurofibromatosis 1
NF2: Neurofibromatosis 2
NMOSD: Neuromyelitis optica spectrum disorder
ON: Optic neuritis
PCA: Posterior cerebral artery
PICA: Posterior inferior cerebellar artery
PNI: Perineural invasion
PSN: Perineural spread
SCA: Superior cerebellar artery
SSFP: Steady state free procession
TOF: Time-of-flight
